# Epigenetic regulator UHRF1 orchestrates proinflammatory gene expression in rheumatoid arthritis in a suppressive manner

**DOI:** 10.1172/JCI150533

**Published:** 2022-06-01

**Authors:** Noritaka Saeki, Kazuki Inoue, Maky Ideta-Otsuka, Kunihiko Watamori, Shinichi Mizuki, Katsuto Takenaka, Katsuhide Igarashi, Hiromasa Miura, Shu Takeda, Yuuki Imai

**Affiliations:** 1 Division of Laboratory Animal Research, Advanced Research Support Center, and; 2 Division of Integrative Pathophysiology, Proteo-Science Center, Ehime University, Ehime, Japan.; 3 Nankai International Advanced Research Institute (Shenzhen Futian), Nankai University, Shenzhen, China.; 4 Laboratory of Instrumental Analysis, School of Pharmacy and Pharmaceutical Sciences, Hoshi University, Shinagawa-ku, Tokyo, Japan.; 5 Department of Bone and Joint Surgery, Ehime University Graduate School of Medicine, Ehime, Japan.; 6 The Center for Rheumatic Diseases, Matsuyama Red Cross Hospital, Ehime, Japan.; 7 Department of Hematology, Clinical Immunology and Infectious Diseases, Ehime University Graduate School of Medicine, Ehime, Japan.; 8 Laboratory of Biofunctional Science, School of Pharmacy and Pharmaceutical Sciences, and; 9 Institute for Advanced Life Sciences, Hoshi University, Shinagawa-ku, Tokyo, Japan.; 10 Division of Endocrinology, Toranomon Hospital Endocrine Center, Minato-ku, Tokyo, Japan.; 11 Department of Pathophysiology, Ehime University Graduate School of Medicine, Ehime, Japan.

**Keywords:** Autoimmunity, Bone Biology, Arthritis, Epigenetics, Rheumatology

## Abstract

Rheumatoid arthritis (RA) is characterized by chronic synovial inflammation with aberrant epigenetic alterations, eventually leading to joint destruction. However, the epigenetic regulatory mechanisms underlying RA pathogenesis remain largely unknown. Here, we showed that ubiquitin-like containing PHD and RING finger domains 1 (UHRF1) is a central epigenetic regulator that orchestrates multiple pathogeneses in RA in a suppressive manner. UHRF1 expression was remarkably upregulated in synovial fibroblasts (SFs) from arthritis model mice and patients with RA. Mice with SF-specific *Uhrf1* conditional knockout showed more severe arthritic phenotypes than littermate controls. *Uhrf1*-deficient SFs also exhibited enhanced apoptosis resistance and upregulated expression of several cytokines, including *Ccl20*. In patients with RA, DAS28, CRP, and Th17 accumulation and apoptosis resistance were negatively correlated with *UHRF1* expression in synovium. Finally, Ryuvidine administration stabilized UHRF1 ameliorated arthritis pathogeneses in a mouse model of RA. This study demonstrated that UHRF1 expressed in RA SFs can contribute to negative feedback mechanisms that suppress multiple pathogenic events in arthritis, suggesting that targeting UHRF1 could be one of the therapeutic strategies for RA.

## Graphical abstract

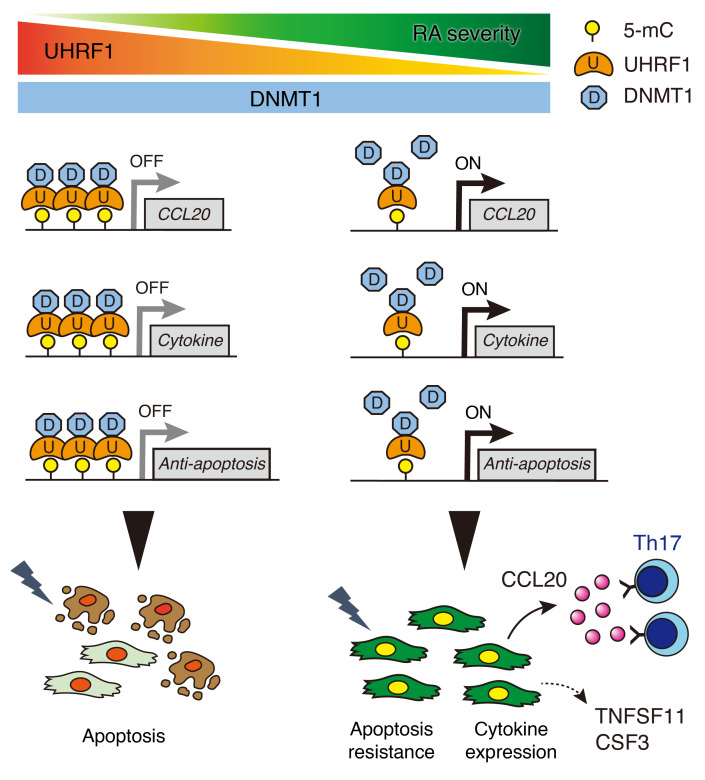

## Introduction

Rheumatoid arthritis (RA) is a systemic autoimmune disease, which has heterogenous symptoms characterized by synovium hyperplasia and joint destruction. Despite the remarkable recent progress in RA therapeutics, including disease-modifying antirheumatic drugs (DMARDs) and biologics that can induce disease remission for a majority of patients with RA, 17% to 23% of patients nonetheless fail to achieve remission after these treatments (1, 2). The number of biologics to treat RA is increasing, but remission rates have not changed (1). In addition, these biologics suppress inflammation by targeting immune system pathways, which increases the risk of serious infection for patients with RA (3). To address these issues, clarification of the molecular mechanisms underlying RA pathogenesis and identification of novel therapeutic targets that do not directly affect immune systems are needed to develop next-generation RA therapeutics.

The etiology of RA is influenced by genetic and environmental factors (4, 5). In the past decade, genome-wide association studies (GWAS) have identified several genetic risk factors in patients with RA (6, 7). However, the spectrum of RA pathogenesis cannot be explained solely based on genetics (8), particularly given the low concordance rate (12%–15%) for RA among monozygotic twins (9), which suggests that epigenetic alterations induced by environmental factors can also contribute to RA pathogenesis.

Epigenetics is one transcriptional regulatory system. Acquired alterations in epigenetics, such as DNA methylation, histone modifications, and chromatin remodeling, contribute to normal biological processes and abnormal cellular behaviors. Indeed, several studies reported that aberrant DNA methylation accounted for progression of various chronic inflammatory diseases (10–12). DNA methylation patterns clearly differ between osteoarthritis (OA) and RA (13, 14). In patients with RA, DNA methylation differs between the early and late phases of RA (15) and between treatment responders and nonresponders (16). RA disease–discordant monozygotic twins also have differential variability in DNA methylation patterns (17, 18). Together, the results from these cohort studies suggest an important role for epigenetic alterations that can affect RA heterogeneity and disease pathology and suggest that epigenetic regulation could be one of the therapeutic strategies and/or a source of biomarkers for patients with RA (19). However, the regulatory mechanisms underlying the establishment of specific DNA methylation signatures in heterogeneous patients with RA are largely unknown. A better understanding of epigenetic pathways in RA is needed to develop therapeutics that can modulate aberrant DNA methylation or identify epigenetic biomarkers for RA.

To identify epigenetic regulators that can contribute to RA pathogenesis, in this study we performed genome-wide gene expression analyses using an arthritis mice model. Our results indicate that ubiquitin-like containing PHD and RING finger domains 1 (UHRF1) could be a central epigenetic regulator in RA. UHRF1 is an essential player in DNA methylation homeostasis through its recognition of hemimethylated DNA and recruitment of DNMT1 to maintain DNA methylation status during DNA replication (20–22). The physiological functions of Uhrf1 have been reported for several cell types, including leukocytes (23–25), chondrocytes (26), and vascular smooth muscle cells (27), but its functions in synovial cells are largely unknown. In this study, we demonstrated that UHRF1 expressed in synovial fibroblasts (SFs) negatively controlled gene expression of multiple exacerbating factors in RA and that UHRF1 stabilization could be an approach to mitigate RA pathogenesis.

## Results

### Upregulation of Uhrf1 expression in arthritis tissue.

**Figure 1:**
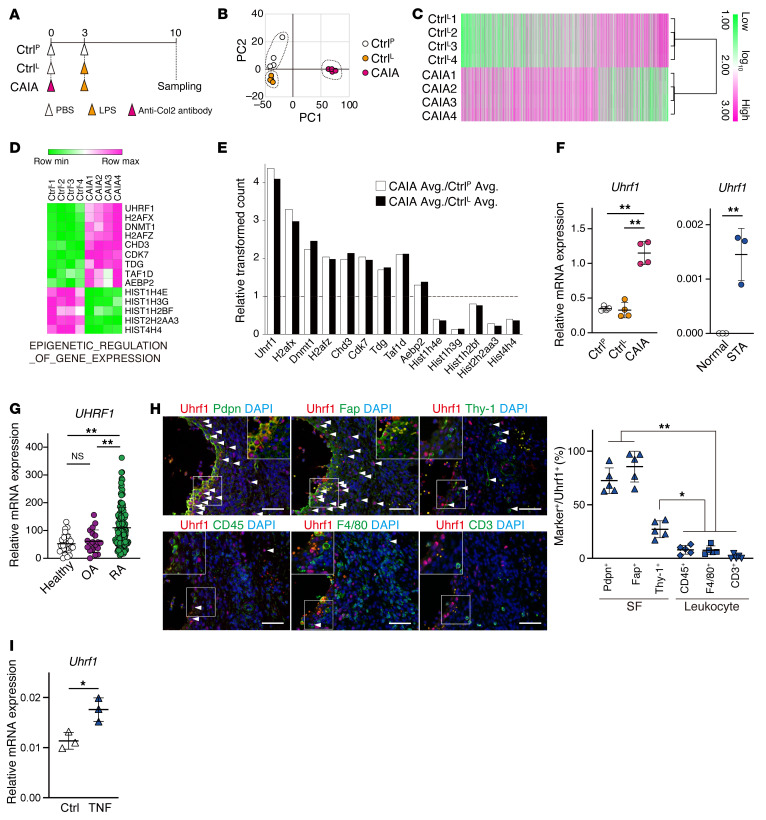
Upregulation of the epigenetic regulator Uhrf1 in arthritis tissue. (**A**) Protocol for analysis of collagen antibody-induced arthritis (CAIA) model. PBS (Ctrl^P^) or LPS (Ctrl^L^) was administered as a control. (**B**) PCA using microarray data obtained from ankle tissue. (**C**) Heatmap of differentially expressed gene probes in ankle tissue (log_2_FC > |1|, *P* < 0.01). Log_10_ transformed read counts are scaled from 1.0 to 3.0. (**D**) Expression of genes related to epigenetic regulation classified by GSEA. Log_10_ transformed read counts are scaled to minimum to maximum values. (**E**) Relative probe counts detected in CAIA ankle compared with Ctrl^P^ or Ctrl^L^ ankles. (**F**) RT-qPCR of *Uhrf1* mRNA expression in CAIA (*n =* 4) and STA (*n =* 3) ankles. (**G**) *UHRF1* mRNA expression in synovium biopsies from healthy individuals, patients with OA, and patients with RA by RNA-Seq. Data are registered in the Gene Expression Omnibus (GEO GSE89408). (**H**) Left, representative images of immunofluorescence staining for Uhrf1 (red); Pdpn, Fap, Thy-1, CD45, F4/80, and CD3 (green); and DAPI (blue) in WT STA ankle tissue. Scale bar: 50 μm. Right, quantification of Uhrf1^+^ marker cells in hyperplastic synovium. Cell number in 1 field per similar region of independent mice was calculated. (**I**) *Uhrf1* mRNA expression in SFs treated with 20 ng/mL Tnf-α for 24 hours. Mean ± SD is shown. *P < 0.05 and **P < 0.01 by ANOVA followed by Tukey’s test in **F** (left), **G**, and **H,** and unpaired *t* test in **F** (right) and **I**. Data in **A**–**F** and **H**–**I** were obtained from 3 to 5 independent experiments.

To identify a candidate epigenetic regulator in RA pathogenesis, we conducted a microarray analysis of gene expression using mRNA obtained from whole ankle tissue from collagen antibody-induced arthritis (CAIA) mice and 2 control mice (Ctrl^P^ and Ctrl^L^; Figure 1A). Principal component analysis (PCA) showed pronounced differences in the gene expression profiles between ankle tissues from CAIA mice and both control mice (Figure 1B). Subsequent microarray analysis revealed that 6155 probes indicated differential expression (4049 and 2106 probes were upregulated and downregulated, respectively) in CAIA ankle compared with Ctrl^L^ ankle (Figure 1C and Supplemental Table 1; supplemental material available online with this article; https://doi.org/10.1172/JCI150533DS1). KEGG pathway analysis showed enrichment of inflammatory- and rheumatoid arthritis–related genes among the upregulated probes in CAIA (Supplemental Figure 1, A and B, and Supplemental Table 1). A subsequent gene set enrichment analysis (GSEA) to classify differentially counted probes in terms of epigenetic regulation (Figure 1D) showed that among the classified gene set, the *Uhrf1* probe count was the most elevated in CAIA ankle compared with both control ankles (Figure 1E). Upregulation of *Uhrf1* mRNA was found not only in CAIA ankle but also in tissue from K/BxN serum transfer arthritis (STA) mice by RT-qPCR (Figure 1F). Analyses of NCBI’s Gene Expression Omnibus (GEO) database for gene expression (GSE89408) revealed that *UHRF1* mRNA was also significantly upregulated in synovium from patients with RA compared with healthy individuals and patients with osteoarthritis (OA), which had similar *UHRF1* mRNA levels (Figure 1G). To assess Uhrf1 localization in synovial tissue, we performed immunofluorescence staining of tissue sections from arthritis model mice. Uhrf1 expression frequently localized in cells that were positive for SF markers (podoplanin [Pdpn], Fap, Thy-1, Col6a1), but was more limited in cells positive for macrophage markers (F4/80, LysM) and nearly absent in CD3^+^ T cells (Figure 1H and Supplemental Figure 1C). Meanwhile, Uhrf1-expressing cells were seen less frequently in healthy synovium (Supplemental Figure 1D). Substantial expression of *Uhrf1* mRNA was observed in primary cultured SFs and synovial macrophages derived from mouse models of both CAIA and STA (Supplemental Figure 1, E and F). *Uhrf1* expression was also significantly elevated by Tnf-α treatment of SFs (Figure 1I). Taken together, these data suggest that Uhrf1 expression levels are dominantly increased in SFs rather than synovial macrophages during pathogenesis of inflammatory arthritis.

### SF-specific deletion of Uhrf1 exacerbates arthritis pathogenesis.

**Figure 2:**
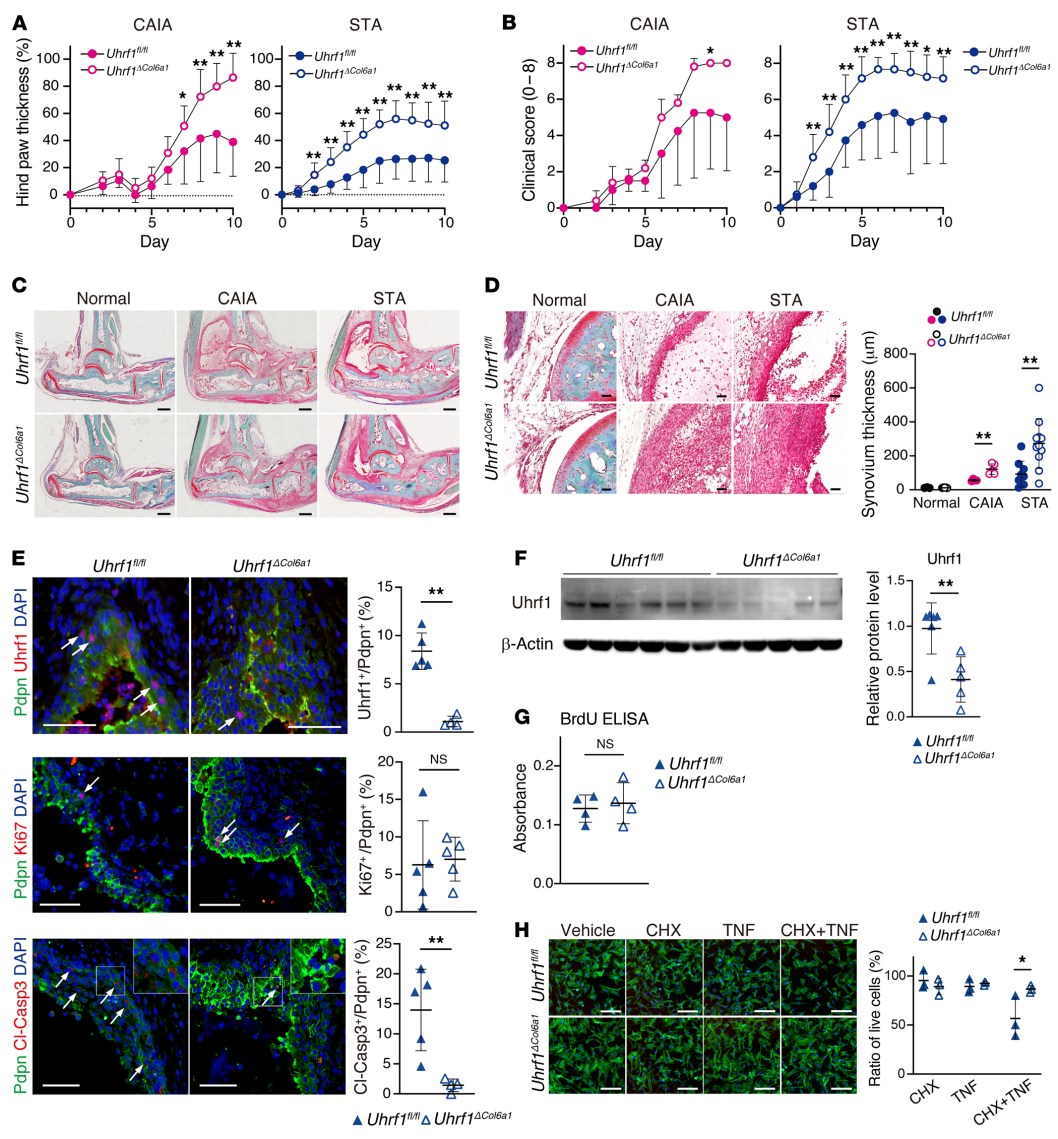
Specific *Uhrf1* depletion in synovial fibroblasts exacerbates arthritis pathogenesis. (**A** and **B**) Development of (**A**) hind paw thickness and (**B**) clinical score in *Uhrf1^fl/fl^* and *Uhrf1^ΔCol6a1^* mice after CAIA (*n =* 4–5) and STA (*n =* 12–15) induction. (**C**) Representative images of safranin O, fast green, and eosin staining of ankle tissue on day 10 after arthritis induction. Scale bar: 500 μm. (**D**) Left, high-magnification images of synovium. Scale bar: 50 μm. Right, quantification of synovium thickness in normal (*n =* 3–5), CAIA (*n =* 4–5), and STA (*n =* 9–11) from *Uhrf1^fl/fl^* and *Uhrf1^ΔCol6a1^* mice. (**E**) Left, immunofluorescence staining for Pdpn (green); Uhrf1, Ki67, and cleaved caspase-3 (Cl-Casp3) (red); and DAPI (blue) in synovium from *Uhrf1^fl/fl^* (*n =* 5) and *Uhrf1^ΔCol6a1^* (*n =* 5) mice. Scale bar: 50 μm. Right, quantification of Uhrf1^+^ Pdpn^+^, Ki67^+^ Pdpn^+^, and Cl-Casp3^+^ Pdpn^+^ cells (arrow) among Pdpn^+^ cells in the synovium region. (**F**) Left, Western blot analysis of primary *Uhrf1^fl/fl^* SFs (*n =* 6) and *Uhrf1^ΔCol6a1^* SFs (*n =* 5) derived from STA ankles. Right, quantification of relative Uhrf1 protein levels. (**G**) BrdU ELISA of SFs from *Uhrf1^fl/fl^* (*n =* 4) and *Uhrf1^ΔCol6a1^* (*n =* 4) mice. (**H**) Left, phalloidin (green) and DAPI (blue) staining of *Uhrf1^fl/fl^* SFs (*n =* 3) and *Uhrf1^ΔCol6a1^* SFs (*n =* 3) derived from STA mice after treatment with 0.5 μg/mL cycloheximide (CHX) and 20 ng/mL Tnf-α for 8 hours. Scale bar: 200 μm. Right, quantification of cell numbers after treatment relative to those for vehicle treatment. Mean ± SD are shown. NS, not significant versus *Uhrf1^fl/fl^*. *P < 0.05 and **P < 0.01 versus *Uhrf1^fl/fl^*, respectively, by unpaired *t* test. All data were obtained from 3–15 independent experiments.

To understand physiological functions of Uhrf1 under arthritis conditions, we next established SF-specific *Uhrf1* conditional knockout mice (*Uhrf1^ΔCol6a1^*). Under normal conditions, the body size of *Uhrf1^ΔCol6a1^* mice was smaller than that for littermate control (*Uhrf1^fl/fl^*) mice, although pathological hallmarks, such as inflammation, were not observed in several different tissues that were tested (Supplemental Figure 2, A–C). Also, *Col6a1-Cre*–driven *Uhrf1* deficiency did not affect Uhrf1 expression in stromal cells of the thymus and lymph nodes (Supplemental Figure 2D). Arthritis was induced in these mice using 2 methods, and development of hind paw swelling was monitored for 10 days. Measured swelling and clinical score for hind paws were significantly more severe in *Uhrf1^ΔCol6a1^* mice than *Uhrf1^fl/fl^* mice (Figure 2, A and B). Morphological analyses showed that hyperplasia of the synovium as well as cartilage and bone destruction were also more severe in *Uhrf1^ΔCol6a1^* mice than in *Uhrf1^fl/fl^* mice (Figure 2, C and D, and Supplemental Figure 2, D–G). Given the detectable presence of Uhrf1 expression in synovial macrophages (Figure 1H and Supplemental Figure 1, E and F) and a previous report that Tnf-α and Ifn-1 expression is regulated by Uhrf1 in macrophages (23, 28), we also established mice with myeloid-specific conditional *Uhrf1* knockout (*Uhrf1^ΔLysM^*). The *Uhrf1^ΔLysM^* mice exhibited no notable phenotypes under either normal conditions or arthritis pathogenesis (Supplemental Figure 3, A–E). Uhrf1 deficiency has also been reported to affect cell cycle and/or apoptosis in certain cell types (29–32). Histologically, we saw no difference in proliferative cell populations of Pdpn^+^ SFs between *Uhrf1^ΔCol6a1^* and *Uhrf1^fl/fl^* mice, whereas the number of apoptotic Pdpn^+^ SFs was significantly reduced in the hyperplastic synovium of *Uhrf1^ΔCol6a1^* mice compared with *Uhrf1^fl/fl^* mice (Figure 2E). To confirm this observation, we isolated primary SFs from arthritis tissue (Figure 2F) and carried out cell proliferation and apoptosis analyses in vitro. Although the BrdU incorporation rate was comparable between *Uhrf1^ΔCol6a1^* and *Uhrf1^fl/fl^* SFs, indicating a similar proliferation rate (Figure 2G), primary SFs derived from *Uhrf1^ΔCol6a1^* mice were significantly more resistant to apoptosis than those from *Uhrf1^fl/fl^* mice (Figure 2H). Collectively, these data demonstrated that Uhrf1 expressed in SFs, but not in synovial macrophages, plays a role in suppressing arthritis pathogenesis through negative feedback mechanisms associated with various arthritis pathologies.

### Uhrf1 depletion induces upregulation of multiple RA-related genes in SFs.

**Figure 3:**
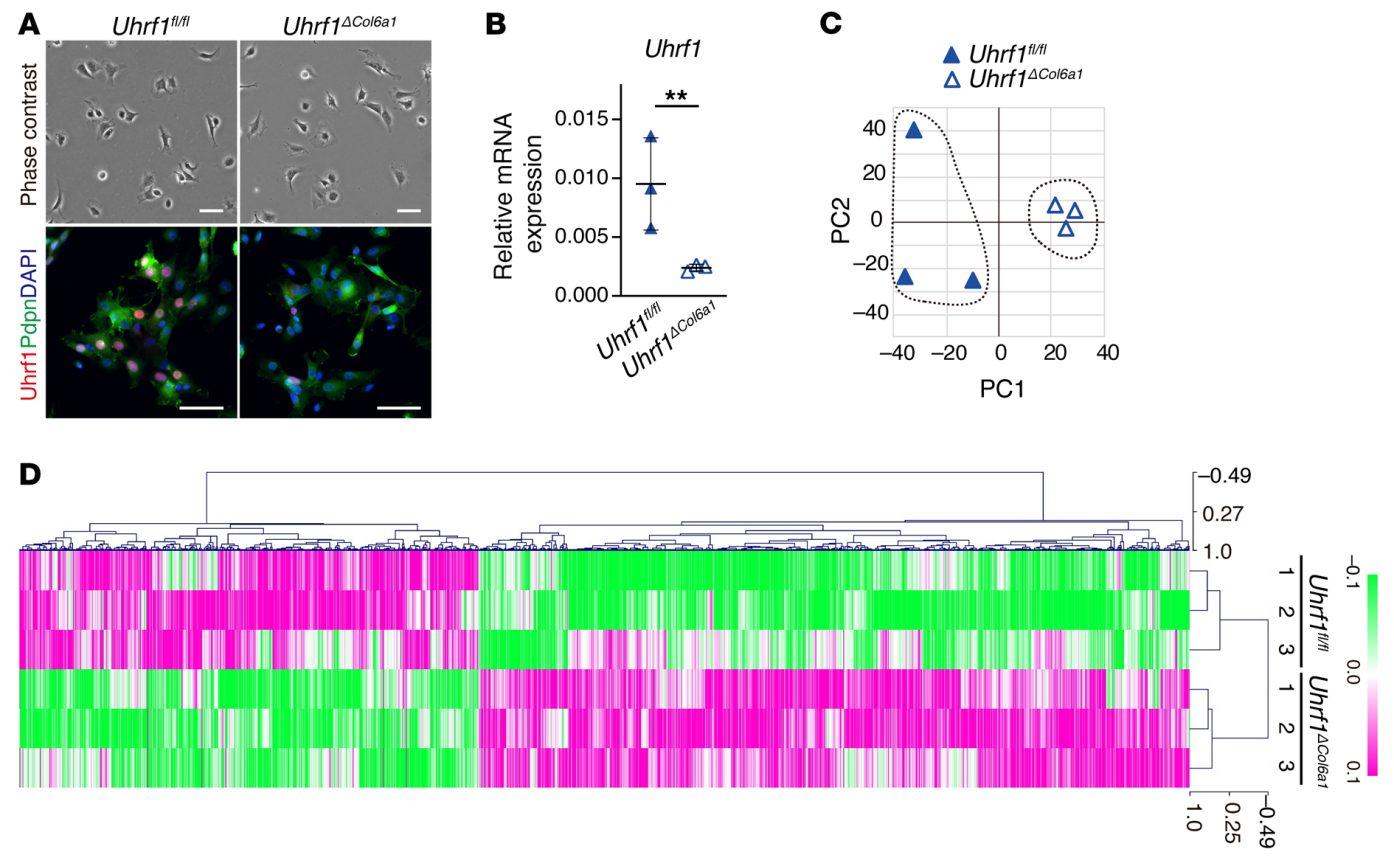
Comparison of gene expression profile between *Uhrf1^fl/fl^* and *Uhrf1^ΔCol6a1^* synovial fibroblasts. (**A**) Top, representative phase contrast images of *Uhrf1^fl/fl^* and *Uhrf1^ΔCol6a1^* SFs derived from STA mice on day 4 after arthritis induction. Bottom, immunostaining for Uhrf1 (red), Pdpn (green), and DAPI (blue). Scale bar: 50 μm. (**B**) RT-qPCR measurement of *Uhrf1* mRNA expression levels in *Uhrf1^fl/fl^* SFs (*n =* 3) and *Uhrf1^ΔCol6a1^* SFs (*n =* 3) derived from STA mice on day 4 after arthritis induction. **P < 0.01. (**C**) PCA analysis of RNA-Seq data. (**D**) Heatmap of differentially expressed genes in *Uhrf1^fl/fl^* and *Uhrf1^ΔCol6a1^* SFs derived from STA mice on day 4 after induction. Log_10_ transformed read counts subtracted from the mean are scaled to –0.1 to 0.1. Data in **A** were technically replicated. Data in **B**–**D** were obtained from 3 independent experiments.

**Figure 4:**
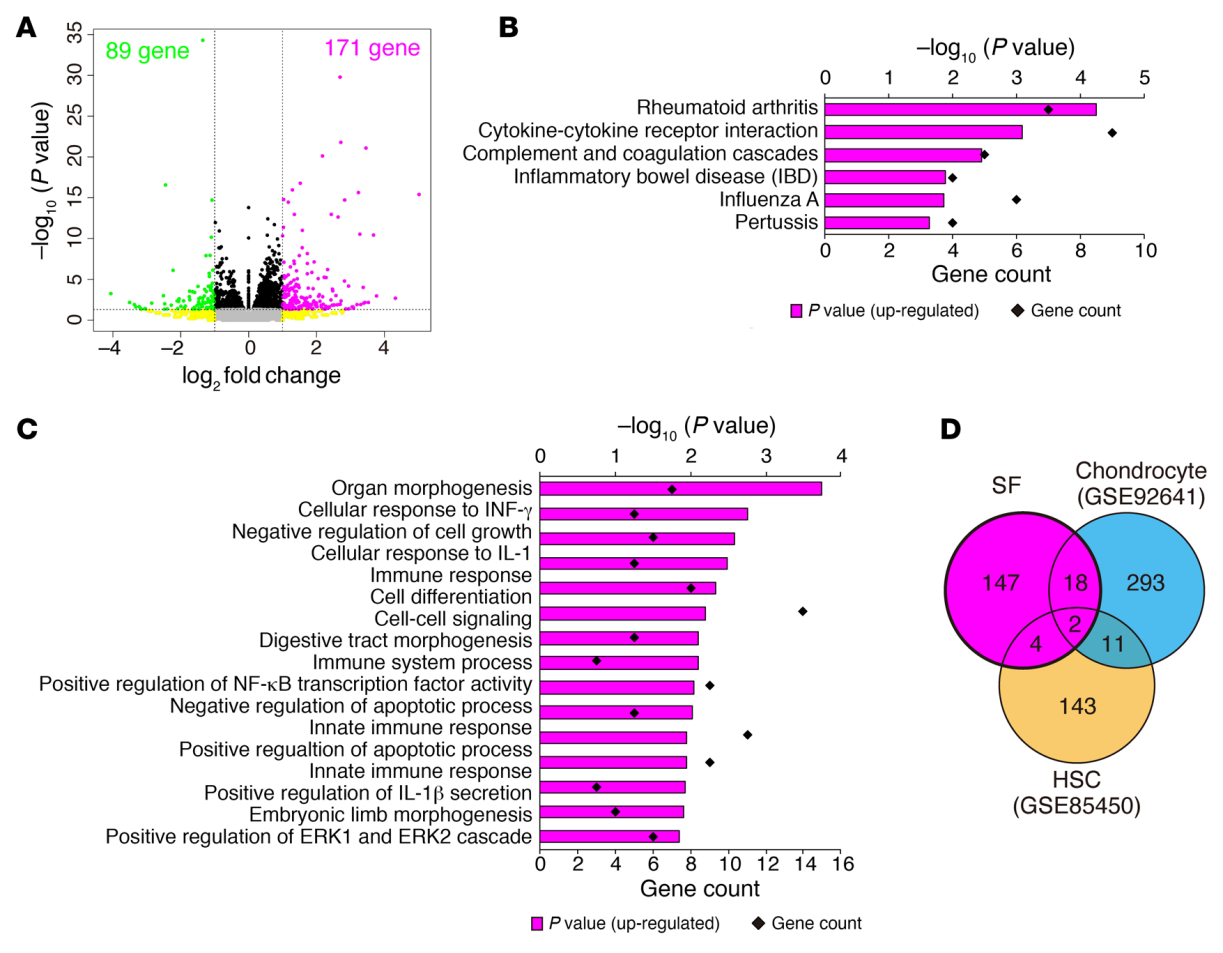
*Uhrf1* depletion induces upregulation of RA-related genes in synovial fibroblasts. (**A**) Volcano plot showing log_2_ fold change (log_2_FC) and statistical significance (*P* value) of differences between *Uhrf1^ΔCol6a1^* and *Uhrf1^fl/fl^* SFs. (**B**) KEGG pathway analysis and (**C**) GO analysis among upregulated genes using DAVID Bioinformatics Resources. Significantly enriched terms are illustrated by gene counts and *P* values. (**D**) Venn diagram comparing upregulated genes (log_2_FC > 1, *P* < 0.05) following *Uhrf1* depletion in SFs, chondrocytes, and hematopoietic stem cells (HSCs) by RNA-Seq analysis of data from this study and data in GEO GSE92641 and GSE85450). Data in **A** were obtained from 3 independent experiments.

Previous reports indicated that Uhrf1 could regulate gene expression genome-wide by regulating DNA methylation (26, 33). To reveal Uhrf1-dependent changes in gene expression, we performed RNA-Seq analysis using SFs obtained from *Uhrf1^fl/fl^* and *Uhrf1^ΔCol6a1^* mice on day 4 in an STA model (Figure 3, A and B). PCA and hierarchical clustering analyses showed an apparently different gene expression profile between SFs isolated from *Uhrf1^fl/fl^* and *Uhrf1^ΔCol6a1^* mice (Figure 3, C and D). Subsequent expression analysis visualized with volcano plots indicated that there were more genes with upregulated expression than those with downregulated expression in SFs from *Uhrf1^ΔCol6a1^* mice versus those from *Uhrf1^fl/fl^* mice (171 genes upregulated and 89 genes downregulated) (Figure 4A and Supplemental Table 2). Kyoto Encyclopedia of Genes and Genomes (KEGG) pathway enrichment analyses revealed that the top 2 pathways for upregulated genes were “Rheumatoid arthritis” and “Cytokine-cytokine receptor interaction,” and that the only pathway having downregulated genes was “Cell adhesion molecules” (Figure 4B, Supplemental Figure 4A, and Supplemental Table 2). Gene Ontology (GO) analyses had significant enrichment in the biological process termed “negative regulation of apoptotic process” among the upregulated genes (Figure 4C, Supplemental Figure 4B, and Supplemental Table 2). Recent studies suggested that biological functions of Uhrf1 could be cell type dependent (24–26). To test this hypothesis, we reanalyzed public RNA-Seq databases (GEO GSE92641, GSE85450) to compare changes in expression of genes that were affected by *Uhrf1* depletion in SFs and other cell types. Differentially expressed genes seen in the presence of *Uhrf1* depletion showed no marked overlap among all cell types considered (Figure 4D, Supplemental Figure 4C, and Supplemental Table 3). These data suggested that *Uhrf1* depletion of SFs upregulates RA- and cytokine-related genes in a cell type–dependent manner.

### Uhrf1 directly regulates mRNA expression of multiple RA-exacerbating factors in SFs via DNA methylation.

**Figure 5:**
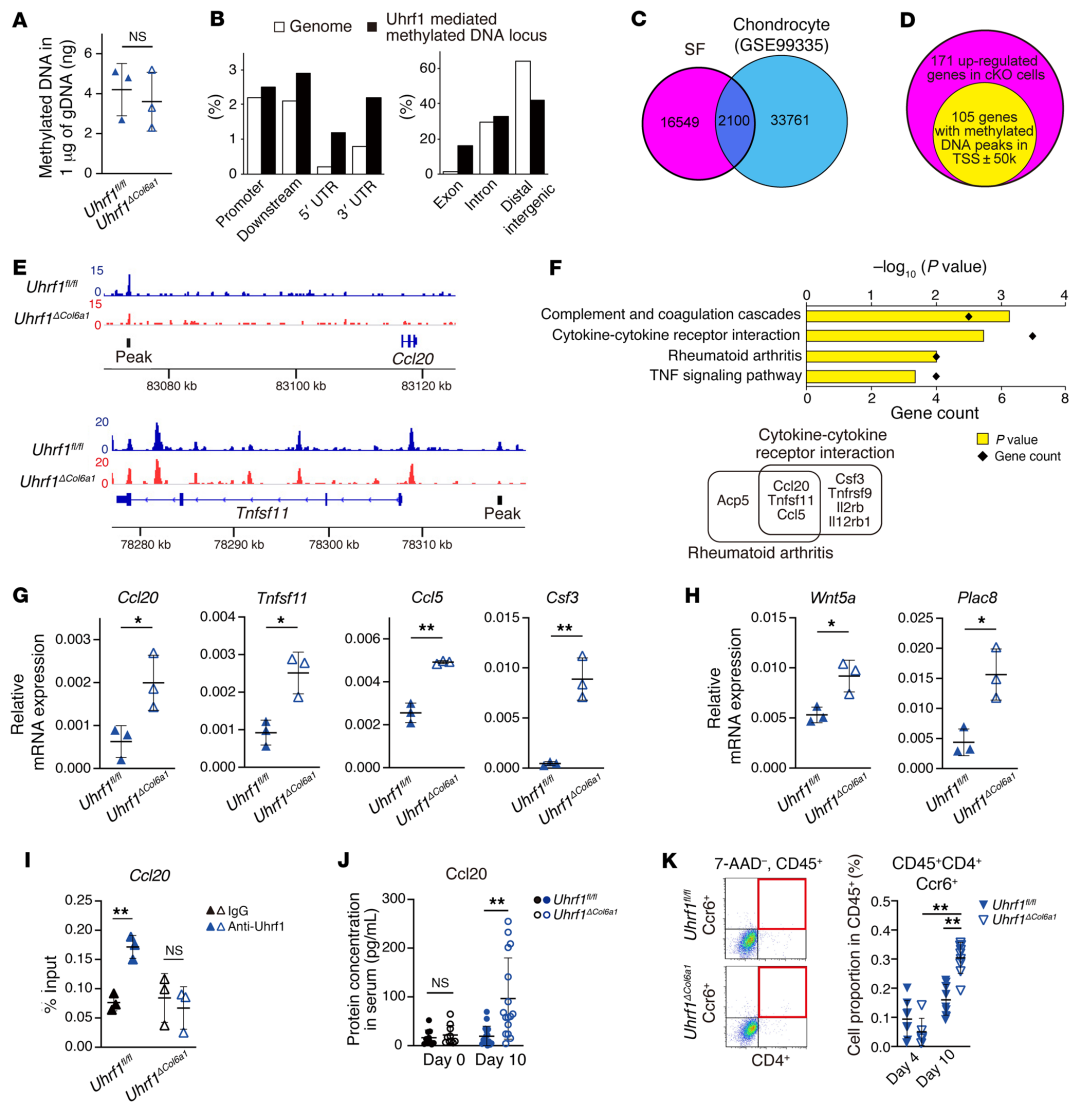
Uhrf1 suppresses expression of multiple genes involved in RA via modulation of DNA methylation. (**A**) Quantification of methylated DNA after enrichment from genome DNA using MBD beads. (**B**) Distribution of Uhrf1-mediated methylated DNA annotated using given intervals and scores with genome features by CEAS. (**C**) Venn diagram to compare Uhrf1-mediated methylated DNA loci between SFs and chondrocytes using MBD-Seq data from this study and GEO GSE99335. (**D**) Venn diagram for 171 genes having upregulated expression in *Uhrf1^ΔCol6a1^* SFs and 105 genes having Uhrf1-mediated methylated DNA peaks within the transcriptional start site region (± 50 kb). (**E**) Representative Uhrf1-mediated methylated DNA peaks visualized by Integrative Genomics Viewer. (**F**) KEGG pathway analysis of 105 upregulated with peaks assigned using DAVID Bioinformatics Resources. Significantly enriched pathways illustrated by gene counts and *P* values. Representative mRNA expression of genes included in the (**G**) KEGG pathways “Rheumatoid arthritis” and “Cytokine-cytokine receptor interaction” and (**H**) GO biological process “Negative regulation of apoptotic process” in SFs from *Uhrf1^fl/fl^* and *Uhrf1^ΔCol6a1^* mice (*n =* 3) as measured by RT-qPCR. (**I**) ChIP-qPCR assay of Uhrf1 for Ccl20 locus in *Uhrf1^ΔCol6a1^* and *Uhrf1^fl/fl^* SFs. (**J**) Quantification of Ccl20 serum levels in *Uhrf1^fl/fl^* and *Uhrf1^ΔCol6a1^* on day 0 (*n =* 10) and day 10 (*n =* 16) after STA induction. (**K**) Left, flow cytometry analysis of the population of Th17 cells (CD45^+^, CD4^+^, Ccr6^+^) in *Uhrf1^fl/fl^* and *Uhrf1^ΔCol6a1^* derived from STA mice on day 4 (*n =* 6–8) and day 10 (*n =* 9–10). Right, quantification of CD45^+^CD4^+^Ccr6^+^ cells among CD45^+^ cells. Mean ± SD shown. NS, not significant versus *Uhrf1^fl/fl^*. *P < 0.05 and **P < 0.01 by unpaired *t* test in **G**–**J** and ANOVA followed by Tukey’s test in **K**. Data in **A**, **G**, and **H** obtained from 3 independent experiments. Data in **B**–**F** obtained from combined read data from 3 independent experiments. Data in **I** were technically replicated 3 times. Data in **J** and **K** obtained from 6–10 independent experiments.

We further analyzed DNA methylation status in SFs by using methyl-CpG-binding domain protein 2 (MBD2) beads to carry out next-generation sequencing on methylated DNA enriched from the whole genome (MBD-Seq). To identify Uhrf1-mediated methylated DNA loci in SFs, we performed peak calling using MACS14 with the locus of downregulated methylation level as peaks. This analysis identified 18,649 Uhrf1-mediated peaks. Cis-regulatory element annotation system (CEAS) analysis showed that the distribution of methylated DNA peaks was altered in *Uhrf1^ΔCol6a1^* SFs against the genome background despite the presence of a nearly commensurate proportion of methylated DNA in the genome (Figure 5, A and B). Moreover, the Uhrf1-mediated methylated DNA peak locus between SFs and chondrocytes (GEO GSE99335) largely did not overlap, supporting the cellular specificity of Uhrf1 function (Figure 5C). To identify genes targeted by Uhrf1 in SFs, we investigated whether Uhrf1-mediated peaks localized at the region surrounding differentially expressed genes in *Uhrf1^ΔCol6a1^* SFs. Among the 171 genes showing Uhrf1-dependent upregulation, 105 genes had peaks in the transcriptional start site ± 50 kb (Figure 5, D and E, and Supplemental Table 2). These 105 genes were highly enriched in KEGG pathways termed “Cytokine-cytokine receptor interaction” and “Rheumatoid arthritis” and included 8 individual genes (Figure 5F and Supplemental Table 2). GO biological process showed a tendency for these genes to be enriched in “negative regulation of apoptotic process,” although this enrichment was not statistically significant (Supplemental Figure 4D and Supplemental Table 2). We used RT-qPCR to validate that *Uhrf1* deficiency altered expression of cytokine- and RA-related genes, including *Ccl20*, *Tnfsf11*, *Ccl5*, and *Csf3* (Figure 5G), and antiapoptosis-related genes, including *Wnt5a* and *Plac8* (Figure 5H). Among the 89 downregulated genes, 39 had peaks in the gene body. Even though none of these genes was enriched in biological process, they may nonetheless contribute to arthritis pathogenesis (Supplemental Figure 4, E and F, and Supplemental Table 2). To further investigate the direct function of Uhrf1, we conducted ChIP-qPCR against a Uhrf1 target locus indicated by a peak by MBD-Seq. We demonstrated direct binding of Uhrf1 upstream of the Ccl20 gene in *Uhrf1^fl/fl^* SFs but not in *Uhrf1^ΔCol6a1^* SFs (Figure 5I). Notably, serum levels of the chemokine Ccl20 were significantly increased in *Uhrf1^ΔCol6a1^* mice compared with *Uhrf1^fl/fl^* mice in late-phase STA (Figure 5J). Ccl20 binds to its unique receptor, Ccr6, to recruit Th17 cells and B cell subsets that have important roles in progression of autoimmune disease (34–36). Flow cytometry analysis showed significantly increased recruitment of CD45^+^CD4^+^Ccr6^+^ (Th17) cells and CD45^+^CD4^–^Ccr6^+^ (expected as a B cell–rich fraction) cells in arthritis tissue from *Uhrf1^ΔCol6a1^* mice in the late but not early phase of STA (Figure 5K and Supplemental Figure 4G). These data suggest that Uhrf1 regulates mRNA expression through control of DNA methylation status of loci for genes that encode multiple exacerbating factors, including Ccl20, that are derived from SFs during arthritis pathogenesis.

### UHRF1 suppresses several processes in RA pathogenesis.

**Figure 6:**
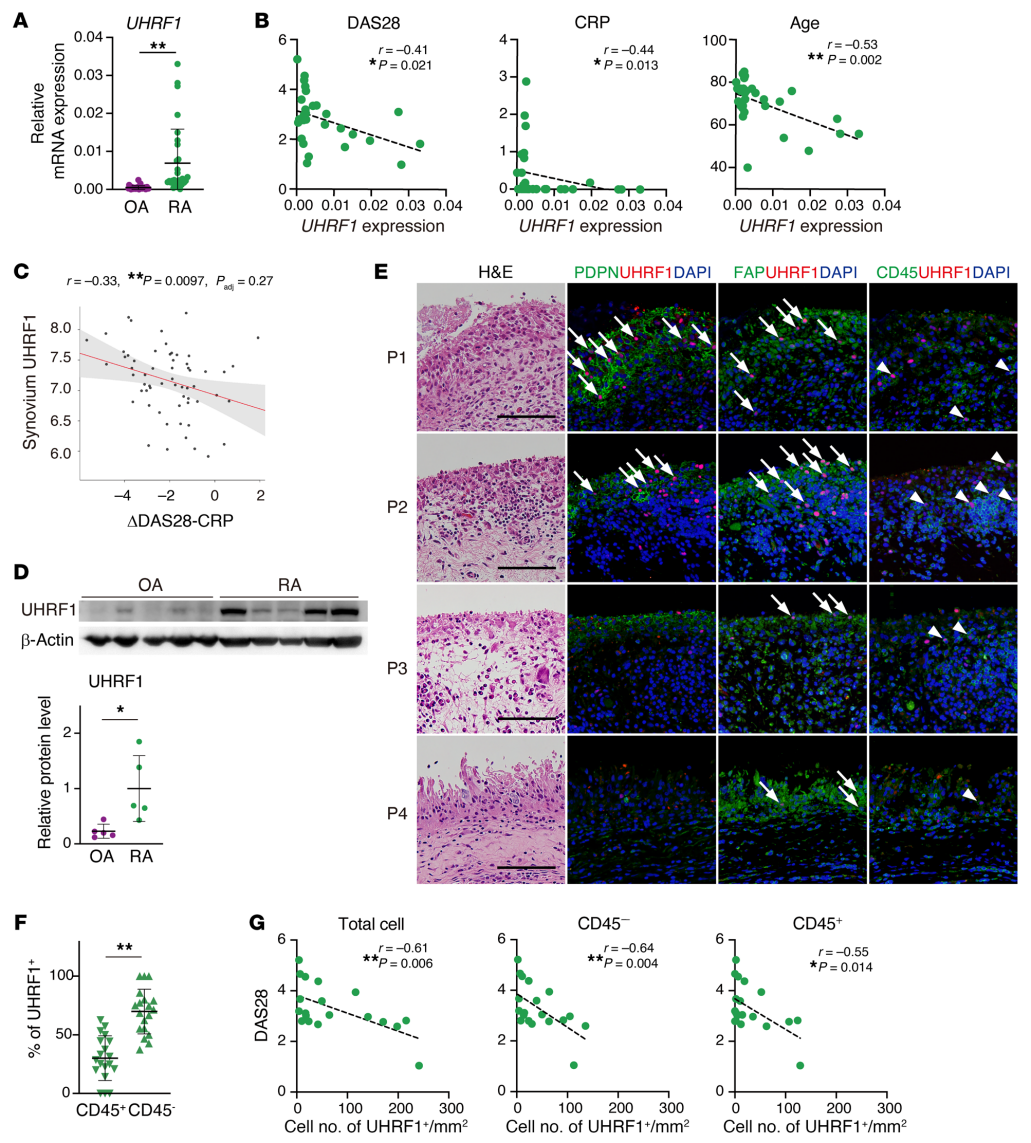
UHRF1 expression is negatively correlated with several RA pathogeneses. (**A**) Expression levels of *UHRF1* and *DNMTs* (*DNMT1*, *DNMT3A*, and *DNMT3B*) mRNA in synovium obtained from patients with OA (*n =* 32) and RA (*n =* 30). (**B**) Spearman’s correlation between *UHRF1* mRNA expression in RA synovium (*n =* 30) and disease activity score 28-CRP (DAS28) as well as levels of C-reactive protein (CRP) and age. (**C**) Correlation of *UHRF1* expression in RA synovium with 6-month response to DMARD treatment measured by ΔDAS28-CRP (https://peac.hpc.qmul.ac.uk/). (**D**) Top, Western blot analysis of OA synovium (*n =* 5) and RA synovium (*n =* 5). Bottom, quantification of relative UHRF1 protein levels. (**E**) H&E staining and immunofluorescence staining for UHRF1 (red); PDPN, FAP, CD45 (green); and DAPI (blue) in specimens from multiple patients with RA (P1–P4). Scale bar: 100 μm. Arrow and arrowhead indicate UHRF1^+^ cells in cells positive for SF markers PDPN and FAP and leukocyte marker CD45, respectively. (**F**) Quantification of UHRF1^+^ cell number in CD45^+^ cells and CD45^–^ cells among total UHRF1^+^ cells (*n =* 19). (**G**) Spearman’s correlation between DAS28 and number of UHRF1^+^ cells per tissue area (*n =* 19). Mean ± SD is shown. *P < 0.05 and **P < 0.01 by Mann-Whitney *U* test in **A** and **D**, and by Wilcoxon signed-rank test in **F**. All data were obtained from 5–32 independent experiments.

To translate our findings to human RA pathogenesis, we next examined the significance of UHRF1 in patients with RA. We collected synovium specimens from patients with OA or RA who underwent knee joint replacement surgery. The patients with RA underwent these operations because of existing symptoms, although almost 90% of patients had been treated with at least one therapeutic agent such as methotrexate and/or biologics. This factor suggests that our cohort included nonresponders to RA treatment. RT-qPCR analysis revealed that mRNA levels of *UHRF1* were significantly elevated in RA synovium relative to those for OA, although the expression level was highly variable among the patients with RA (Figure 6A). Meanwhile, mRNA expression levels of DNA methyltransferases (*DNMT1*, *DNMT3A*, and *DNMT3B*) were similar between OA and RA samples, which is consistent with a previous report (ref. 37 and Supplemental Figure 5A), and suggests that RA-specific aberrant DNA methylation and/or RA heterogeneity of disease severity are dependent on UHRF1 expression level. Several clinical parameters, including disease activity score 28 (DAS28), C-reactive protein (CRP), and age, were negatively correlated with *UHRF1* mRNA expression levels in RA synovium, whereas there was no correlation with levels of *DNMT1*, *DNMT3A*, and *DNMT3B* (Figure 6B and Supplemental Figure 5, B–E). In addition, we assessed the correlation between *UHRF1* expression and shift of DAS28 by treatment with DMARDs for 6 months using a publicly available RNA-Seq data set of RA synovium (https://peac.hpc.qmul.ac.uk/). We observed significant negative correlation between levels of *UHRF1* expression and treatment responsiveness for DAS28 (Figure 6C), suggesting that sufficient expression levels of UHRF1 are needed to respond to RA treatment. To further investigate the suppressive function of UHRF1 for RA pathogenesis, we validated specimens based on UHRF1 protein levels. Compared with OA synovium, UHRF1 protein levels also varied, but overall were significantly upregulated in synovium from patients with RA (Figure 6D). To determine localization of UHRF1 expression in synovium tissue, immunofluorescence staining was conducted using multiple specimens. UHRF1 expression was frequently detected in cells that were positive for SF markers (PDPN and/or FAP) in patients with RA, but not those with OA, although PDPN expression was absent in some RA specimens (Figure 6E and Supplemental Figure 5F). In parallel, UHRF1 also localized in partial CD45^+^ leukocytes (Figure 6E). Cell count analysis revealed that the overall frequency of UHRF1-expressing cells was significantly higher in CD45^–^ stromal cells than in CD45^+^ cells (Figure 6F), suggesting that UHRF1 was mainly expressed in SFs in patients with RA and in the mouse model of inflammatory arthritis. Additional histological analyses indicated that the frequency of UHRF1 detection was not associated with hyperplasia of the synovial lining layer (Figure 6E). Thus, we assessed the correlation between DAS28 and UHRF1 frequency. DAS28 was negatively correlated with UHRF1 frequency per area not only in total cells and CD45^–^ cells but also in CD45^+^ cells (Figure 6G). A previous study showed that UHRF1 expression was essential for proliferation of Tregs (25). Since functional deficiency of Tregs has been proposed as a mechanism for progression of several autoimmune diseases, including RA (38), a negative correlation of CD45^+^ frequency with DAS28 would be reasonable. These results suggested that both mRNA and protein levels of UHRF1 were increased because of RA but not OA pathogenesis. On the other hand, insufficient levels of UHRF1 could further exacerbate RA.

**Figure 7:**
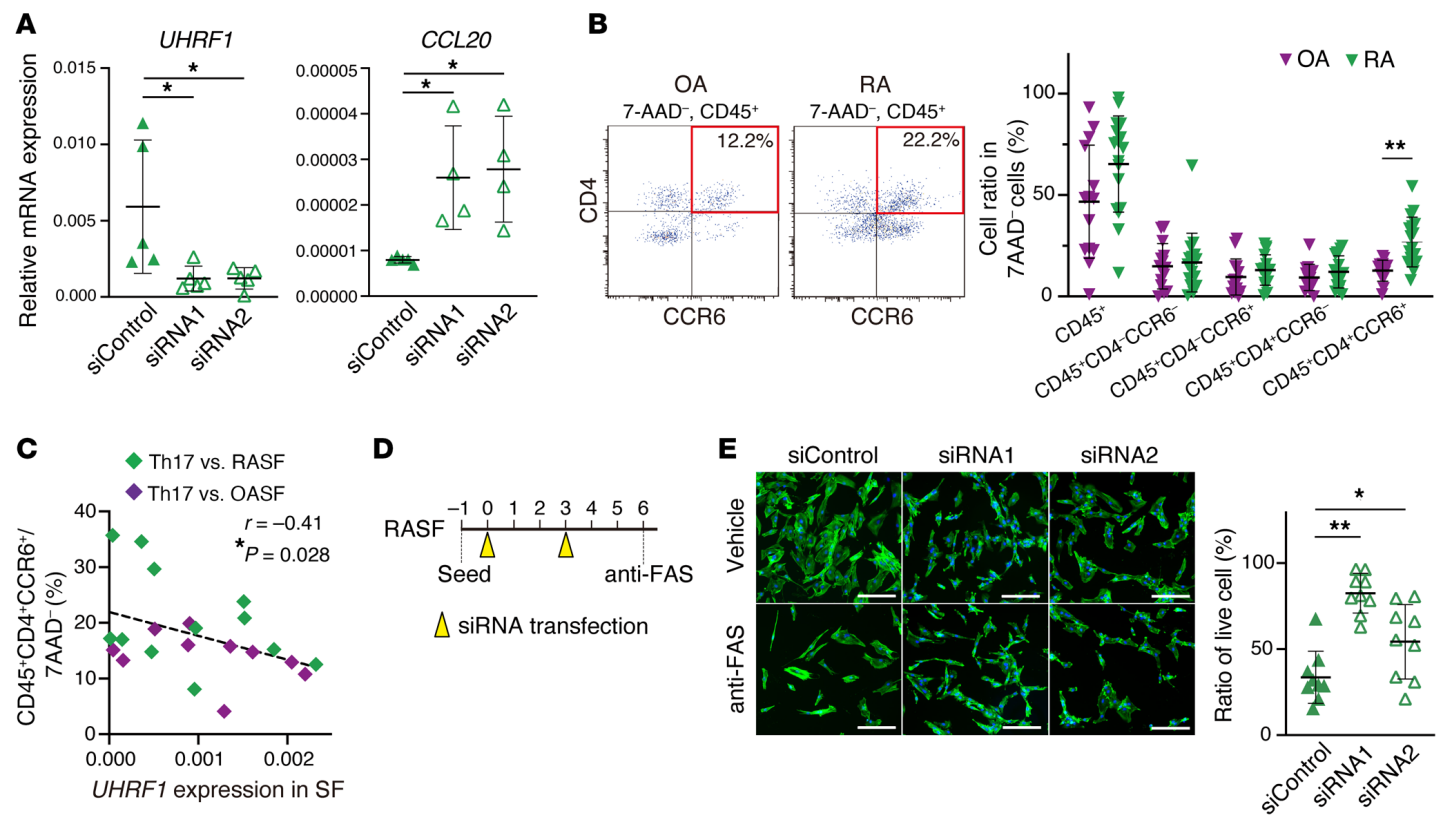
UHRF1 negatively regulates CCL20 expression and apoptosis resistance in RA. (**A**) mRNA expression levels of *UHRF1* and *CCL20* in RASFs transfected with UHRF1 siRNA (*n =* 4–5). (**B**) Left, flow cytometry to measure proportion of Th17 cells (CD45^+^CD4^+^CCR6^+^) in OA (*n =* 14) and RA (*n =* 21) synovium tissue. Right, quantification of total CD45^+^ cells, CD45^+^CD4^–^CCR6^–^ cells, CD45^+^CD4^–^CCR6^+^ cells, CD45^+^CD4^+^CCR6^–^ cells, and CD45^+^CD4^+^CCR6^+^ cells among 7-AAD^–^ cells. (**C**) Spearman’s correlation between proportion of Th17 cells and *UHRF1* mRNA expression level in OASFs (*n =* 10) and RASFs (*n =* 12) obtained from synovium of the same patients. (**D**) Schematic protocol of consecutive *UHRF1* knockdown and experimental apoptosis induction in RASFs. (**E**) Left, phalloidin (green) and DAPI (blue) staining of RASFs transfected twice with UHRF1 siRNA (*n =* 9) after treatment with 0.5 μg/mL anti-FAS antibody for 16 hours. Scale bar: 200 μm. Right, quantification of cell numbers after apoptosis induction relative to that for vehicle treatment. Mean ± SD is shown. *P < 0.05 and **P < 0.01 by ANOVA followed by Tukey’s test in **A** and **E**, and Mann-Whitney *U* test in **B**. All data were obtained from 4 to 21 independent experiments.

To test whether UHRF1 regulates expression of notable genes, including cytokine- and RA-related pathways in both murine SFs and SFs from patients with RA (RASFs), *UHRF1* knockdown was performed in SFs from patients with OA (OASFs) and RASFs. *UHRF1* mRNA suppression was significantly associated with upregulation of *CCL20* mRNA expression in RASFs but not OASFs, supporting the notion that regulation of RA-related gene expression by UHRF1 is dependent on RA pathogenesis (Figure 7A and Supplemental Figure 5G). Focusing on CCL20, we used flow cytometry to examine the proportion of Th17 cells in RA and OA synovia. Although we observed no large difference in the population of other leukocytes between OA and RA synovium samples, the population of Th17 cells was significantly elevated in RA (Figure 7B and Supplemental Figure 5H). To confirm that accumulation of Th17 cells is regulated by UHRF1 expression in human SFs, we then investigated the correlation between the frequency of Th17 cells and *UHRF1* mRNA expression in SFs in synovium samples from the same patients. We found that the Th17 frequency was indeed negatively correlated with *UHRF1* mRNA expression levels in human SFs (Figure 7C). In addition, consecutive knockdown of *UHRF1* mRNA resulted in the resistance to FAS-induced apoptosis in RASFs (Figure 7, D and E), similar to that seen for murine SFs lacking *Uhrf1* (Figure 2H). Collectively, these data demonstrated that UHRF1 affects arthritis pathogenesis in a suppressive manner by regulating Th17 recruitment and apoptosis of SFs both in murine models of arthritis and in human RA.

### Uhrf1 stabilization ameliorates arthritis pathogenesis.

**Figure 8:**
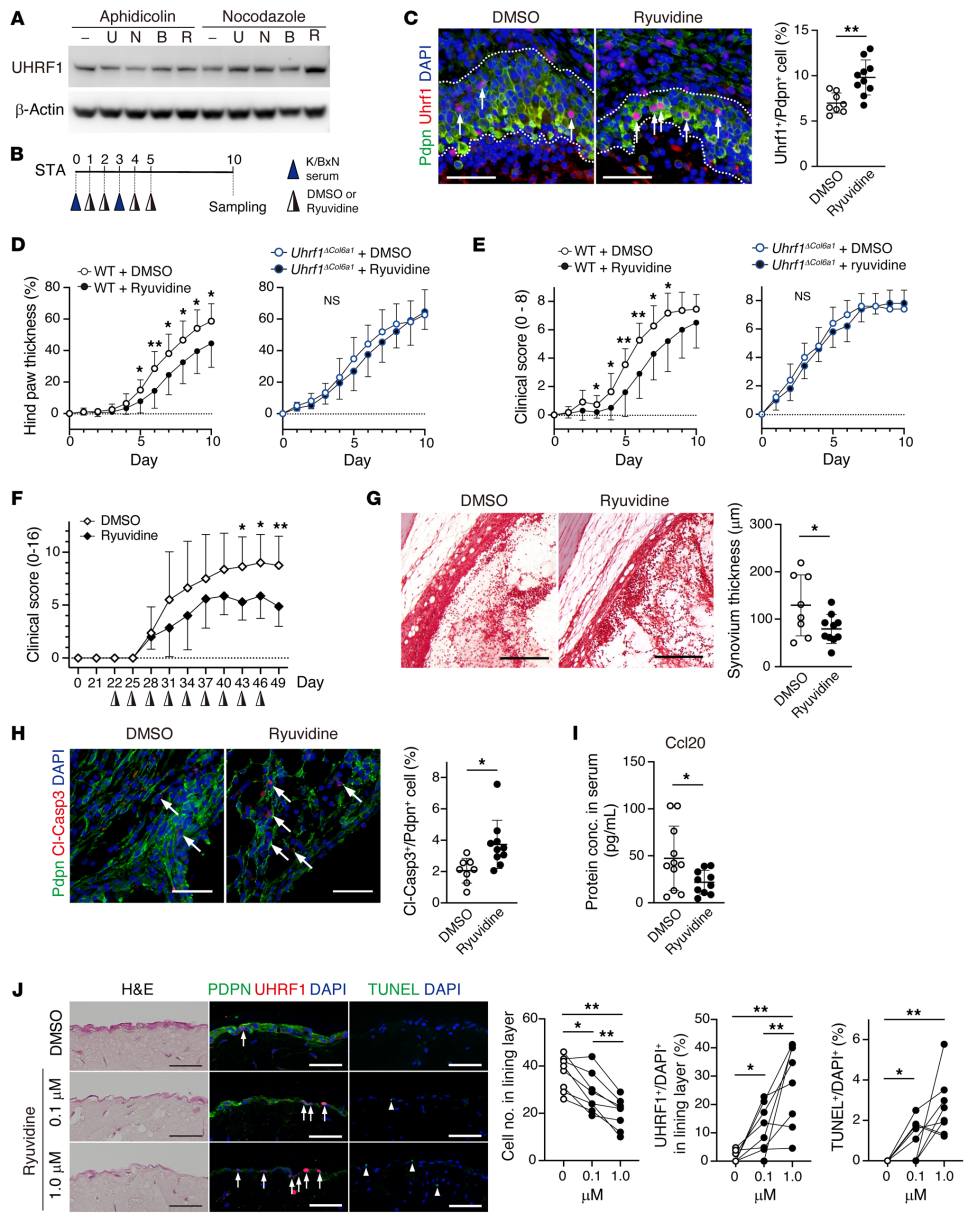
Uhrf1 stabilization attenuates arthritis pathogenesis. (**A**) Western blot analysis of UHRF1 expression in HEK293 cells. Cell cycle was synchronized with aphidicolin (G_1_/S phase) or nocodazole (G_2_/M phase) before cells were treated with UNC0379 (U), NSC663284 (N), BVT948 (**B**), or Ryuvidine (R). (**B**) Protocol to assess efficacy of Ryuvidine in STA. (**C**) Immunofluorescence staining for Uhrf1 (red), Pdpn (green), and DAPI (blue) in WT STA with or without Ryuvidine treatment. Scale bar: 50 μm. Quantification of Uhrf1^+^ Pdpn^+^ (arrow) per population of Pdpn^+^ cells. Development of (**D**) hind paw thickness and (**E**) clinical score in WT and *Uhrf1^ΔCol6a1^* mice with or without Ryuvidine injection after STA induction. (**F**) Development of clinical score in DBA/1 mice with or without Ryuvidine injection after collagen-induced arthritis induction. (**G**) Representative safranin O, fast green, and eosin staining in WT STA. Scale bar: 200 μm. Quantification of synovium thickness in WT STA after Ryuvidine treatment. (**H**) Immunofluorescence staining for Cl-Casp3 (red), Pdpn (green), and DAPI (blue) in WT STA with or without Ryuvidine treatment. Scale bar: 50 μm. Quantification of Cl-Casp3^+^ Pdpn^+^ (arrow) per population of Pdpn^+^ cells. (**I**) Quantification of Ccl20 serum levels in WT STA after Ryuvidine treatment. (**J**) H&E staining and immunofluorescence staining for UHRF1 (red); PDPN, TUNEL (green); and DAPI (blue) in RASF organoids with or without Ryuvidine treatment. Scale bar: 50 μm. Quantification of cell number in lining layer, UHRF1^+^ among DAPI^+^ (arrow) cells in the lining layer and TUNEL^+^ cells per DAPI^+^ (arrowhead) cells in the field. WT + DMSO; *n =* 8–12, WT + Ryuvidine; *n =* 10–12, *Uhrf1^ΔCol6a1^* + DMSO; *n =* 5, *Uhrf1^ΔCol6a1^* + Ryuvidine; *n =* 5, DBA/1 + DMSO; *n =* 8, DBA/1 + Ryuvidine; *n =* 7, organoid culture; *n =* 8. Mean ± SD. *P < 0.05 and **P < 0.01 versus DMSO by unpaired *t* test in **C**–**I**, and ANOVA followed by Tukey’s test in **J**. Data in **A** were technically replicated. Data in **B**–**J** obtained from 5–12 independent experiments.

The above-mentioned results demonstrated that preservation of UHRF1 expression could attenuate RA pathogenesis. Although the precise molecular mechanisms underlying degradation of UHRF1 are largely unclear, a recent study reported that methylation of UHRF1 protein by methyltransferase SET8 (also called SETD8, PR-SET7, and KMT5A) promotes ubiquitination-dependent protein degradation of UHRF1 (39). In addition, SET8 inhibitors (UNC0379, NSC663284, BVT948, and Ryuvidine) are reported to reduce methylation levels of other proteins (40, 41). Thus, we initially assessed whether these inhibitors can stabilize UHRF1 protein levels using HEK293 cells. Cell-cycle synchronization revealed that UHRF1 was the protein that showed the greatest degree of stabilization following Ryuvidine treatment in the G_2_/M phase, which is known as the UHRF1 degradation phase (ref. 39 and Figure 8A). We also administered Ryuvidine to STA model mice (Figure 8B). Immunofluorescence staining of WT tissue samples showed that sustainable Uhrf1 expression was achieved by Ryuvidine treatment in vivo after STA induction (Figure 8C). Incipient administration of Ryuvidine significantly delayed exacerbation of arthritis phenotypes in WT STA model mice compared with mice treated with DMSO (Figure 8, D and E). In addition, in a mouse model of autoimmune arthritis, collagen-induced arthritis, Ryuvidine administration significantly improved arthritis pathogenesis (Figure 8F). However, Ryuvidine treatment did not ameliorate symptoms in *Uhrf1^ΔCol6a1^* STA model mice (Figure 8, D and E). These data suggested that the beneficial effect of Ryuvidine treatment on autoimmune arthritis was dependent on UHRF1 stabilization. Histopathological features, such as hyperplasia and apoptosis resistance in the synovium, as well as Ccl20 serum levels, were also significantly reduced by Ryuvidine treatment on day 10 in the WT STA model (Figure 8, G–I). To investigate whether UHRF1 stabilization could have therapeutic value for RA, the beneficial effects of Ryuvidine for synovial hyperplasia were analyzed ex vivo. We generated 3D-cultured organoids using RASFs with or without Ryuvidine treatment. Histologically, hyperplasia of the synovial lining layer was significantly and dose-dependently inhibited by Ryuvidine, and these effects were associated with enhanced frequency of UHRF1 expression and apoptosis rate (Figure 8J). Taken together, these results indicate that stabilization of UHRF1 protein is a potential therapeutic strategy for patients with RA.

## Discussion

Epigenetic alterations are one potential mechanism to promote RA heterogeneity, and treatments that target proteins involved in epigenetic changes could be one of the therapeutic strategies for patients with RA, particularly those who do not respond to current treatments (19, 42). Indeed, DNA methyltransferase inhibitors, such as 5-azacitidine, have been approved and used as epigenetic drugs to treat various types of cancers (43). The therapeutic effect of 5-azacitidine has also been tested in an arthritis model and RA-derived cells (44, 45). The results suggested that 5-azacitidine could improve arthritis pathogenesis by upregulating expression of the antiinflammatory cytokine *IL-10* and inhibiting IgG1 production (44, 45). On the other hand, 5-azacitidine treatment produces multiple side effects, such as cytotoxicity, thrombocytopenia, and aberrant spermatogenesis (46). To achieve application of epigenetic drugs for RA therapeutics, regulatory mechanisms associated with induction of RA-specific aberrations in DNA methylation should be elucidated and controlled.

In this study, we identified UHRF1 as a suppressive modulator of multiple exacerbating factors in RA (Supplemental Figure 6). UHRF1 is known to contribute to maintenance of DNA methylation through recruitment of DNMT1 during cell division (20–22). In addition, several studies indicated that UHRF1 interacts with de novo DNA methyltransferases DNMT3A and DNMT3B as well as DNMT1 (47) via mechanisms that are not yet clear. Thus, UHRF1 might play a role in both proliferating and nonproliferating cells. Previous studies reported that global DNA methylation levels in RASFs were lower than that in OASFs (48) and that OA and RA have differentially methylated loci (13, 14, 37). Previous single-cell RNA-Seq data from the Accelerating Medicines Partnership (AMP) RA did not present sufficient *UHRF1* mRNA in any types of synovial cells (https://immunogenomics.io/ampra/). However, since our analysis using synovium tissue showed substantive UHRF1 expression in both mRNA and protein levels, the lack of UHRF1 detection in AMP was probably due to read depth. Also, in the present study, we observed higher and more diverse expression levels of UHRF1 but not *DNMTs* in RA compared with OA, and these levels were negatively correlated with DAS28 in patients with RA. These data indicate that UHRF1 has a central epigenetic role to induce RA-specific DNA methylation patterns that could help suppress exacerbation of symptoms. Therefore, UHRF1 may serve as a biomarker for disease severity and/or one of possible determining criteria for RA heterogeneity. The function of UHRF1 in maintenance of DNA methylation is thought to be mediated through DNMT1, although whether suppressive functions of Uhrf1 in arthritis can be completely mediated through Dnmt1 is unclear. Here, we attempted to generate SF-specific *Dnmt1* conditional knockout mice (*Dnmt1^ΔCol6a1^*), but most *Dnmt1^ΔCol6a1^* mice died before reaching adulthood (data not shown). This is a limitation of this study.

Previous studies showed that UHRF1 expression was regulated by transcription factors, such as SP1, E2F1, E2F8, and FOXM1, for the cell cycle and NF-κB during inflammation (49, 50). In our experiments, *Uhrf1* mRNA expression was upregulated in Tnf-α–stimulated SFs (Figure 1I), and mRNA levels for *E2f1*, *E2f8*, *Foxm1*, *Nfkb1*, *Nfkb2*, *Relb*, and *Rel* were upregulated in CAIA ankle tissue in microarray analysis (Supplemental Table 1). Increased expression levels of UHRF1 might be a hyperplastic hallmark and/or a consequence of chronic inflammation in RA pathogenesis and play a role in negative feedback of these pathogeneses. Meanwhile, in some members of our RA cohort, UHRF1 expression levels were comparatively low. Our results explained that decreased amounts of UHRF1 induced aggravation of RA pathogenesis and/or inhibited beneficial effects of medications, but the mechanisms that contribute to low UHRF1 levels in some patients with RA are unclear. UHRF1 depletion has been reported not only to reduce DNA methylation but also to induce cellular DNA damage (51, 52). Since mRNA expression of *Cdkn1a* and *Cdkn2a*, which are representative senescence marker genes, is regulated by UHRF1 in lymphocytes (24, 25) and cancer cells (53), the cellular phenotypes caused by UHRF1 depletion can resemble those of cellular senescence. Therefore, deficient UHRF1 expression might be related to cellular senescence in RASFs. Our data, which includes a Uhrf1 deficiency–induced antiapoptotic phenotype (Figure 2F), showing increased *Cdkn2a* mRNA levels in *Uhrf1^ΔCol6a1^* SFs (Supplemental Table 2) and a negative correlation between UHRF1 expression levels and age (Figure 6B), support this possibility. Lack of UHRF1 in RASFs might induce expression of several cytokines, such as SASP (senescence-associated secretory phenotype), that have effects that are independent of direct alterations in DNA methylation. Further experiments are required to clarify the relationship between UHRF1 and cellular senescence in patients with RA.

Our integrative analyses of the transcriptome and methylome of synovial tissue from a murine arthritis model and patients with RA showed that CCL20 is a common UHRF1 target gene among cytokine-, RA-, and antiapoptosis-related genes. However, a role for other genes (*CSF3*, *TNFSF11*, *CCL5*, *TNFRSF9*, *IL2RB*, *IL12RB1*, *ACP5*, *WNT5A*, and *PLAC8*) was not validated in transient *UHRF1* knockdown of RASFs (data not shown). These data indicate that regulation of the expression of specific gene(s) by UHRF1 is dependent on species and/or arthritis types since previous reports showed that DNA methylation patterns differ between arthritis types, such as OA and RA (13, 14). Our data also showed that different pathologies related to cartilage degradation were associated with Uhrf1 depletion in CAIA and STA mice (Supplemental Figure 2D). However, our findings showed that arthritis phenotypes that are dependent on UHRF1 expression levels were largely common between humans and mice. Thus, we assessed whether Uhrf1 stabilization can improve arthritis pathogenesis. We identified Ryuvidine as a candidate chemical to stabilize UHRF1 protein. Indeed, Ryuvidine treatment could ameliorate arthritis pathogenesis in model mice (Figure 8, D–I) and hyperplasia in RASF organoids (Figure 8J). A previous report postulated that UHRF1 stabilization by Ryuvidine is mediated via inhibition of SET8 (39), which can induce protein methylation of not only UHRF1 but also histone H4 and p53 (40, 41), suggesting that Ryuvidine treatment may affect other biological processes. Although further preclinical studies will be needed to develop UHRF1 stabilization as an RA therapeutic strategy, our results provide a basis for investigation of a new therapeutic strategy that has efficacy toward different pathways than those targeted by existing agents, including methotrexate or biologics.

Collectively, our finding that Ryuvidine treatment ameliorated arthritis provides support for the ability of UHRF1 stabilization to inhibit expression of multiple exacerbating factors in RA. These findings could contribute to a basis for exploration of alternative therapeutic approaches, especially for patients who do not respond to existing treatments.

## Methods

### Antibodies.

The primary antibodies used in this study included mouse monoclonal antibody against human and mouse UHRF1 (hmUhrf1; Santa Cruz Biotechnology sc-373750); rabbit monoclonal antibody against human UHRF1 (hUHRF1; Abcam, ab194236); rat monoclonal antibody against mouse Pdpn (mPdpn; Wako, 015-24111); mouse monoclonal antibody against human PDPN (hPDPN; BioLegend, 916606); rat monoclonal antibody against mouse Fap (mFap; R&D Systems, MAB9727); rabbit polyclonal antibody against human FAP (hFAP; Bioss, bs-5758R); rabbit monoclonal antibody against human and mouse Thy-1 (Cell Signaling Technology, 13801); rabbit polyclonal antibody against mouse CD45 (mCD45; Abcam, ab10558); mouse monoclonal antibody against human CD45 (hCD45; BioLegend, 304002); rabbit monoclonal antibody against mouse F4/80 (Cell Signaling Technology, 70076); rat monoclonal antibody against human and mouse CD3 (Bio-Rad, MCA1477T); rabbit polyclonal antibody against GPF (Cell Signaling Technology, 598); rabbit polyclonal antibody against human and mouse cleaved caspase-3 (Cell Signaling Technology, 9661); rabbit monoclonal antibody against human and mouse Ki-67 (Abcam, ab16667); and mouse monoclonal antibody against human and mouse β-actin (MBL, M177-3). The secondary antibodies used included Alexa Fluor 488–conjugated goat anti-rat IgG; Alexa Fluor 488–conjugated goat anti-rabbit IgG; Alexa Fluor 568–conjugated goat anti-mouse IgG1; Alexa Fluor 568–conjugated goat anti-rat IgG; Alexa Fluor 568–conjugated goat anti-rabbit IgG (Molecular Probes); and HRP-conjugated goat anti-mouse IgG (DAKO). Flow cytometry antibodies used included FITC-conjugated rat antibody against mouse CD45 (BioLegend, 30-F11); PE-conjugated rat antibody against mouse CD4 (BioLegend, GK1.5); Alexa Fluor 647–conjugated rat antibody against mouse Ccr6 (BD Biosciences, 140706); APC-conjugated mouse antibody against human CD45 (Miltenyi Biotec, 5B1); FITC-conjugated mouse antibody against human CD4 (BioLegend, OKT4); and PE-conjugated mouse antibody against human CCR6 (BioLegend, G034E3). Mouse monoclonal antibody against human FAS (MBL, CH-11, SY-001) was used to induce functional apoptosis.

### Human synovial specimens.

Human synovial specimens were obtained from patients with OA or RA who underwent knee joint replacement surgery at the Ehime University Hospital and Matsuyama Red Cross Hospital. For histological analysis, synovial tissues were fixed with 4% PFA for 6–8 hours and then embedded in paraffin. To obtain OASFs and RASFs, synovial tissues were minced and treated with 1 mg/mL collagenase type IV (Sigma-Aldrich) in DMEM GlutaMax (Gibco) supplemented with 10% FBS (Sigma-Aldrich) and 1% antibiotic-antimycotic solution (anti-anti, Gibco) for 6 to 8 hours before filtration through a 40 μm cell strainer (Falcon). Filtered cells were seeded in culture dishes, and the most adherent cells were considered to be OASFs and RASFs. Human SFs were used within passage 5. Human SFs were cultured in DMEM GlutaMax supplemented with 10% FBS and 1% anti-anti solution and cultured at 37°C in a humidified atmosphere of 5% CO_2._

### Mice.

Uhrf1 mutant knockout-first mice (B6Dnk B6N-Uhrf1^tm1a(EUCOMM)Wtsi^/Ieg; strain EM:04084) were obtained from the European Mouse Mutant Archive (EMMA). ACTB-Flpe mice (B6.Cg-Tg(ACTFLPe)9205Dym/J; strain 005703), R26^NZG^ mice (FVB.Cg-*Gt(ROSA)26Sor^tm1(CAG-lacZ,-EGFP)Glh^*/J; strain 012429), and *LysM-Cre* (B6.129P2-Lyz2^tm1(cre)Ifo^/J; strain 004781) mice were obtained from The Jackson Laboratory. *Col6a1-Cre* (54) (B6. Cg-Tg(Col6a1-Cre) 1Gkl/Flmg) mice were provided by George Kollias (Biomedical Sciences Research Centre, Athens, Greece). KRN mice (55) were provided by Christophe Benoist and Diane Mathis at Harvard Medical School, Boston, MA, USA. C57BL/6 (WT), and NOD/ShiJcl mice were obtained from CLEA Japan. DBA/1 JJmsSlc mice were obtained from SLC Japan.

To generate Uhrf1-floxed mice (*Uhrf1^f/fl^*), knockout-first mice were crossed with ACTB-Flpe mice. *Uhrf1^f/fl^* mice were crossed with Cre mice to generate *Col6a1-Cre Uhrf1^fl/fl^* (*Uhrf1^ΔCol6a1^*) mice and *LysM-Cre Uhrf1^fl/fl^* (*Uhrf1^ΔLysM^*) mice, respectively. To generate cell type–specific reporter mice, R26^NZG^ mice were crossed with *Col6a1-Cre* mice and *LysM-Cre* mice, respectively. To generate K/BxN mice, KRN mice, which were backcrossed with C57BL/6 mice, were crossed with NOD/ShiJcl mice. All mice were housed in a specific pathogen–free facility under climate-controlled conditions with a 12-hour light/12-hour dark cycle and were provided with water and standard diet (MF, Oriental Yeast) ad libitum.

### Arthritis model mice studies.

At postnatal 7 weeks, female mice were subjected to CAIA, K/BxN STA, or collagen-induced arthritis. CAIA induction was conducted as previously described (56). Briefly, 5 mg anti-collagen 2 monoclonal antibody cocktail (Chondrex, Redmond) was administered on day 0, followed by 50 μg LPS i.p. on day 3. For STA induction, 50 μL K/BxN serum was i.p. administered on days 0 and 3. For collagen-induced arthritis induction, DBA/1 mice were immunized with 100 μg chicken type II collagen (Sigma-Aldrich) emulsified in complete Freund’s adjuvant containing 0.5 mg/mL *Mycobacterium tuberculosis* by intradermal injection at the base of the tail, followed by a booster injection of collagen in incomplete Freund’s adjuvant 21 days after the first injection. A single 0.8 μg/g dose of Ryuvidine in DMSO and corn oil (16 μL/g body weight) was i.p. administered on days 1, 2, 4, and 5 for STA and every 3 days after the second immunization for collagen-induced arthritis. We monitored the development of swelling by measuring hind paw thickness (ratio of average increased thickness of both hind paws) and assigned clinical scores: 0, no erythema or swelling; 1, erythema and swelling in up to 2 joints; 2, erythema and swelling in more than 2 joints or mild swelling of ankle; 3, moderate swelling of tarsals and ankles; 4, severe swelling of tarsals and ankles (the sum score of both hind paws for CAIA and STA; 4 paws for collagen-induced arthritis). For histological analysis, mice were anesthetized and then rapidly euthanized with reflux flow of PBS. Ankle tissues were obtained and fixed overnight with 4% PFA, followed by decalcification with 0.5 M EDTA for 2 weeks. The samples were embedded in paraffin after dehydration and 6–7 μm thick paraffin sections were cut with a microtome (RM2255, Leica Biosystems). The sections were deparaffinized and used for safranin O-fast green-hematoxylin staining and tartrate-resistant acid phosphatase (TRAP) staining (TRAP staining kit, Wako).

### Murine synovial cell studies.

Primary cultures of SFs and synovial macrophages were obtained from swollen ankle tissues from CAIA and K/BxN STA mice, respectively, as previously described (56). Briefly, mice on 4 and 10 days after arthritis induction had blood removed by reflux flow of PBS under anesthesia, and the swollen ankles were harvested by dislocation and treated with 1 mg/mL collagenase type IV (Sigma-Aldrich) in DMEM GlutaMax supplemented with 10% FBS and 1% anti-anti for 1–2 hours with shaking before filtration with a 40 μm cell strainer (Falcon). To obtain SFs, filtered cells were cultured for 1 hour on a culture dish precoated with collagen (type I-C, Nitta gelatin) in DMEM GlutaMax supplemented with 10% FBS and 1% anti-anti solution. Nonadherent cells were then removed. To obtain synovial macrophages, filtered bulk cells were cultured for 1 to 2 weeks before fibroblastic cells were gently detached by trypsin treatment. Cells remaining on the dish were used as synovial macrophages. Primary SFs and synovial macrophages were cultured in DMEM GlutaMax supplemented with 10% FBS and 1% anti-anti solution before use in some experiments. The cells used for experiments were from passages 0 to 3. All cells were cultured at 37°C in a humidified atmosphere of 5% CO_2._

Primary SFs were seeded in 96-well plates at 1 × 10^4^ cells/well. To test the rate of cellular proliferation, a BrdU assay was performed using a cell proliferation ELISA kit (Roche Molecular Biochemicals). BrdU solution was added, and cells were incubated for an additional 2 hours at 37°C. After fixation of cells, BrdU incorporation was measured according to the manufacturer’s instructions. To assess apoptosis, cells were treated with or without 25 ng/mL Tnf-α and 0.5 μg/mL cycloheximide (CHX) for 8 hours. After fixation, the cells were stained with Alexa Fluor 488–conjugated phalloidin (Thermo Fisher Scientific) and DAPI for 30 minutes at room temperature. The number of nuclei per field was automatically counted using ImageJ (NIH). To calculate the percentage of living cells, the number of nuclei in the treated cells was divided by that for vehicle-treated cells.

Immunocytochemical staining was performed as previously described (56). Briefly, cells were fixed with 4% PFA for 5–10 minutes and then permeabilized with 0.5% Triton X-100 PBS for 5 minutes before blocking with 1% BSA and 0.02% Triton X-100 PBS. Primary antibodies were added at 1:100 (anti-hmUhrf1, mPdpn) and incubated for 1 hour at room temperature. After washing, secondary antibodies were incubated with 5 μg/mL DAPI for 30 minutes at room temperature.

### Synovial organoid culture.

RASFs were suspended in ice-cold Matrigel (Corning) at 2 × 10^6^ cells/mL, and 25 μL droplets of the cell suspension were placed on culture dishes coated with poly-HEMA (Sigma-Aldrich). After incubation for 30 minutes at 37°C, the micromass was cultured in culture medium for 1 week, followed by treatment with 0, 0.1, and 1.0 μM Ryuvidine for 1 week with a medium change every 2 to 3 days. The micromass was then fixed with 4% PFA for 2 hours before embedding in paraffin.

### Immunofluorescence staining.

Immunofluorescence staining was performed as previously described (26, 56). Briefly, deparaffinized 4–5 μm thick sections were boiled at 85°C to 90°C for 60 minutes with 0.05% citraconic acid solution (ImmunoSaver; Wako) to retrieve antigens. After blocking for 60 minutes (Blocking One Histo, Nacalai Tesque), the sections were incubated overnight at 4°C with primary antibodies diluted in immune reaction enhancer solution (Can Get Signal) at 1:50 (anti-hmUhrf1), 1:100 (anti-hUHRF1, mPdpn, hPDPN, mFap, hFAP, Thy-1, mCD45, hCD45, F4/80, CD3, cleaved-caspase-3, Ki67), or 1:500 (anti-GFP). After washing with PBS, 5 μg/mL secondary antibodies with DAPI were reacted for 60 minutes at room temperature. To detect apoptotic cells histologically, TUNEL was performed (Roche). After blocking, deparaffinized sections were reacted with fluorescein-conjugated dUTP for 60 minutes at room temperature according to the manufacturer’s instructions.

### Flow cytometry.

To produce single-cell suspensions, murine ankle tissue and human synovia samples were digested with collagenase type IV. Bulk cells were stored at –80°C before use. Thawed cell suspensions were seeded in culture dishes and preincubated in medium overnight at 37°C. Nonadherent cells were used for flow cytometry analysis. Murine cells were stained with anti-CD45, CD4, Ccr6, and 7-AAD (7-amino-actinomycin D; Thermo Fisher Scientific). Human cells were stained with anti-CD45, CD4, CCR6, and 7-AAD. Expression of cell surface markers was evaluated using FACSAria (BD Biosciences) and Gallios (Beckman Coulter) instruments. Data were analyzed using FlowJo software (Treestar Inc.).

### Cell-cycle synchronization.

UNC0379, NSC663284, BVT948, and Ryuvidine were purchased from Cayman Chemical as candidate chemical agents that stabilize the UHRF1 protein. For synchronization at the G_1_/S border, HEK293 cells (ATCC) were treated with 1 μg/mL aphidicolin for 6 hours followed by treatment with 1 μg/mL aphidicolin with or without 1 μM of the indicated chemical agent for 16 hours. For synchronization in the G_2_/M phase, cells were treated with 50 ng/mL nocodazole with or without 1 μM of the indicated chemical agent for 16 hours.

### Real time RT-PCR.

Total RNA was extracted with Isogen (Nippon Gene) and RNeasy spin column kits (Qiagen). First-strand cDNA was synthesized from the total RNA using PrimeScript RT Master Mix (Takara Bio) and subjected to real-time RT-PCR using TB Green Premix Ex Taq II (Takara Bio) with Thermal Cycler Dice (Takara Bio) according to the manufacturer’s instructions. Gene expression levels were normalized relative to those of the housekeeping gene *RPLP0* (*Rplp0*). Primer sequences for each gene are listed in Supplemental Table 4.

### Western blotting.

Cells were washed with PBS and dissolved in RIPA buffer with protease inhibitor cocktail (Nacalai Tesque). Whole-cell extracts were separated by SDS-PAGE and transferred to PVDF membranes, which were blocked with 3% BSA in TBS with 0.5% skim milk and 0.05% Triton X-100 (TBST). The membranes were then incubated with anti-hmUhrf1 antibody (1:250) and anti–β-actin antibody (1 μg/mL) overnight at 4 °C. After washing with TBST, HRP-conjugated secondary antibody (1:5000) was bound for 1 hour at room temperature. Immunoreactive signals were detected with ECL prime (GE Healthcare) and an ImageQuant LAS 4000 instrument (GE Healthcare).

### siRNA experiments.

siRNA specific for UHRF1 was purchased from Thermo Fisher Scientific. The sequence for the siRNA construct targeting the UHRF1 gene was UHRF1-1 (5′-CUGCUUUGCUCCCAUCAAU-3′), UHRF1-2 (5′-GCCAUACCCUCUUCGACUA-3′). MISSION siRNA Universal Negative Control (Sigma-Aldrich) was used as a control siRNA. To analyze gene expression in OASFs and RASFs, 5 × 10^4^ cells were transfected with 2 pmol siRNA using an electroporation apparatus (Neon, Invitrogen) as previously described (26). Cells were used 48 hours after transfection. To analyze apoptosis resistance, 1 × 10^4^ cells/cm^2^ cells were transfected with 3 pmol siRNA using Lipofectamine RNAiMax (Thermo Fisher Scientific) according to the manufacturer’s instructions. On day 3 after the first transfection, siRNA was retransfected and the cells were then cultured for another 2 days. The transfected cells were reseeded in 96-well plates (2.5 × 10^3^ to 3.0 × 10^3^ cells/well) and 1 day later were incubated with 0.5 μg/mL anti-FAS antibody for 16 hours to induce functional apoptosis. After fixation, the cells were stained with Alexa Fluor 488–conjugated phalloidin (Thermo Fisher Scientific) and DAPI for 20 minutes at room temperature. The number of nuclei per field was automatically counted using ImageJ (NIH). To calculate the percentage of living cells, the number of nuclei seen for treated cells was divided by the number of nuclei seen for vehicle-control cells.

### ELISA.

Ccl20 protein concentration in mice serum was measured using a Mouse Ccl20 ELISA kit (R&D Systems). Absorbance at 450 nm was measured using a FluxStation3 (Molecular Devices) according to the manufacturer’s instructions.

### ChIP assay.

Chromatin isolation was performed using a ChIP-IT High Sensitivity kit (Active Motif). Briefly, primary SFs were obtained from STA ankles. Approximately 1 × 10^6^ SF cells were cross-linked by 1% formaldehyde in medium for 15 minutes at room temperature, followed by quenching with 125 mM glycine for 5 minutes. After homogenization, the cell suspensions were sonicated with a Covaris S220. After centrifugation, the supernatant was reacted with 4 μg anti-hmUhrf1 (sc-373750) and normal mouse IgG (sc-2343) antibodies at 4°C overnight with rotation. The immune complexes were precipitated using protein G agarose beads at 4°C for 3 hours on a rotator. ChIP reactions were transferred into a filtration column and then eluted. After reversing the cross-linking, DNA was purified using a purification column. Purified DNA was used for qPCR using primers listed in Supplemental Table 4.

### Microarray analysis.

Total RNA was extracted from whole ankle tissue using Isogen and RNeasy Mini kit (Qiagen). The total RNA was used to generate cRNA according to the GeneChip (Thermo Fisher Scientific) protocol. After reverse transcription by SuperScript II (Invitrogen) and conversion into double-stranded cDNA, a MinElute Reaction Cleanup kit (Qiagen) was used for purification. The purified double-stranded cDNA was transcribed and labeled in vitro using a BioArray HighYield RNA Transcript Labeling kit (Enzo Life Sciences). The labeled cRNA was then purified using RNeasy Mini kit (Qiagen). The purified cRNAs were hybridized to GeneChip Mouse Genome 430 2.0 arrays and washed and stained in a GeneChip Fluidics Station. The phycoerythrin-stained arrays were scanned to obtain digital image files, which were then analyzed using GeneChip Operating Software (Affymetrix). The microarray data set was deposited in GEO under accession number GSE167190.

### RNA-Seq analysis.

Murine SFs were isolated from swollen ankle tissue on 4 days after K/BxN STA induction. After culturing for 1 day, high-quality total RNA was obtained from the SFs using RNeasy spin column kits and verified using an Agilent 2100 Bioanalyzer. RNA-Seq analysis was performed as previously described (26, 56). RNA-Seq libraries were prepared using an Illumina TruSeq Stranded mRNA LT Sample Prep kit according to the manufacturer’s instructions. The libraries were subsequently validated for an average size of approximately 311 to 328 bp using a 2100 Bioanalyzer and an Agilent DNA1000 kit. Sequencing of paired-end reads (75 bp) was performed with a MiSeq Reagent kit V3 150 cycle on a MiSeq system (Illumina). Sequence data were mapped on the mouse genome (mm10) using TopHat (57) and analyzed using Cufflinks (58). The RNA-Seq data set was deposited in GEO under accession number GSE166746.

### MBD-Seq analysis.

MBD-Seq was performed to analyze genome-wide methylated and/or nonmethylated DNA regions as previously described (26). Briefly, methylated DNA was enriched by MBD2-mediated precipitation and subjected to next-generation sequencing. Highly methylated DNA regions were identified by sequence reads mapped on the reference genome. Extracted DNA from murine SFs was sonicated with a Covaris sonicator to obtain approximately 300 bp fragments. MBD2-mediated enrichment of methylated DNA was performed using the methylated DNA enrichment kit EpiXplore (Takara Bio) according to the manufacturer’s instructions. The amount of enriched methylated DNA in 1 μg total DNA was measured using a Quantus Fluorometer (Promega). Libraries for MBD-Seq analysis were prepared using a QIAseq Ultralow Input Library kit (Qiagen) according to the manufacturer’s instructions and validated for an average size of approximately 300 to 700 bp using a TapeStation and the Agilent High Sensitivity D1000 ScreenTape kit. Each experiment was biologically replicated at least 3 times. Sequencing of paired-end reads (75 bp) was performed using the MiSeq Reagent kit V3 150 cycle on a MiSeq system (Illumina) and mapped on the mouse genome (mm10) using CLC Genomics Workbench (Qiagen). The MBD-Seq data set was deposited in GEO under accession number GSE166747.

### Analysis of sequencing data.

Differentially expressed genes having expression levels that were significantly increased or decreased by more/less than twice/half that of the control were extracted for further analyses. Hierarchical cluster analysis and PCA were carried out using MeV (59), and GO analyses were performed with DAVID Bioinformatics Resources 6.8 (60) and GSEA (61). For MBD-Seq, peak calling was performed using MACS14 (62), and integrative analyses were done using Cistrome Analysis Pipeline (http://cistrome.org/ap/) as previously described.

### Statistics.

Two-tailed unpaired Student’s *t* test and Mann-Whitney *U* test with GraphPad Prism 8 were used to analyze differences between 2 groups. One-way ANOVA followed by post hoc Tukey’s test with GraphPad and SPSS (IBM) were applied to compare multiple groups. Spearman’s rank correlation coefficient with SPSS was applied to assess the strength and direction of monotonic association between paired data. For all graphs, data are represented as the mean ± SD. Statistical significance was accepted when *P* values were less than 0.05.

### Study approval.

Experiments involving human samples were approved by the IRB of Ehime University (1802018) and Matsuyama Red Cross Hospital (674). All patients provided informed written consent to participate in the study. Experiments involving animals were approved by the Animal Experiment Committee of Ehime University and were performed in accordance with Ehime University Guidelines for Animal Experiments (37A1-1*16).

## Author contributions

NS, K Inoue, and YI planned the study and designed the experiments. NS, K Inoue, MIO, and K Igarashi generated the microarrays, and NS, MIO, and K Igarashi performed MBD-Seq and assisted with data interpretation. NS performed all other experiments with support and advice from KT and the other authors. NS, HM, SM, KW, and YI collected human specimens. NS and YI wrote the manuscript with input from ST and the other authors.

## Supplementary Material

Supplemental data

Supplemental table 1

Supplemental table 2

Supplemental table 3

Supplemental table 4

## Version 1 04/26/2022

In-Press Preview

## Version 2 06/01/2022

Electronic publication
