# Supplementary material for: Epigenetic regulator UHRF1 orchestrates proinflammatory gene expression in rheumatoid arthritis in a suppressive manner
Source: J Clin Invest. 2022 Jun 1;132(11):e150533. doi: 10.1172/JCI150533 (PMC9151705; doi:10.1172/JCI150533)

Supplementary Information for

Epigenetic regulator UHRF1 suppressively orchestrates pro-inflammatory gene expression in rheumatoid arthritis.

Noritaka Saeki, Kazuki Inoue, Maky Ideta-Otsuka, Kunihiko Watamori, Shinichi Mizuki, Katsuto Takenaka, Katsuhide Igarashi, Hiromasa Miura, Shu Takeda, Yuuki Imai*

*Corresponding author. E-mail: y-imai@m.ehime-u.ac.jp

The pdf file includes:

Figure legend of Supplemental Figure 1 to 6

Supplemental Figure 1 to 6

Supplemental Figure 1: Uhrf1 is highly expressed in arthritis-derived SF.

(**A** and **B**) KEGG pathway analysis of (A) up- and (B) down-regulated gene probes in CAIA ankle tissue by microarray analysis. The top 10 terms that showed significant enrichment are illustrated with gene counts and *P* values. (**C** and **D**) Immunofluorescent staining for Uhrf1 (Red), GFP (Green) and DAPI (Blue) in (C) *Col6a1Cre; Rosa26-EGFP* and *LysMCre; Rosa26-EGFP* after STA induction and (D) Uhrf1 (Red), Fap (Green), F4/80 (Green) and DAPI (Blue) in *WT* normal ankle tissue. Arrows indicate Uhrf1^{+} GPF^{+} and Uhrf1^{+} Fap^{+} cells. Scale bar represents 50 μm. (**E** and **F**) RT-qPCR measurement of mRNA expression of SF markers (*Cdh11, Col6a1, Tnfsf11*), macrophage markers (*Cd68, Emr1, Tnfrsf11a*) and *Uhrf1* in primary cultures of SF and SM derived from (E) CAIA ankle and (F) STA ankle. Mean±SD is shown. *N.S.*; not significant. ** indicates $P<0.01$ by unpaired *t*-test. Data in (C) and (D) were obtained from more than 2 independent experiments and technically replicated. Data in (E) and (F) were obtained from 4-5 independent experiments.

Supplemental Figure 2: SF-specific *Uhrf1* depletion has pathological effects but not under healthy conditions.

(**A**) Macroscopic images of SF-specific *Uhrf1* knockout mice (*Uhrf1*$^{\Delta Col6a1}$) and littermate control mice (*Uhrf1*$^{fl/fl}$). (**B**) Quantification of body weight of 7-week-old *Uhrf1*$^{fl/fl}$ and *Uhrf1*$^{\Delta Col6a1}$ mice (n=23). (**C**) Hematoxylin-eosin (H.E.) and Masson's trichrome (M.T.) staining of intestine, liver, lung and kidney tissue from *Uhrf1*$^{fl/fl}$ and *Uhrf1*$^{\Delta Col6a1}$ mice under healthy conditions. Scale bar represents 100 μm. (**D**) Immunofluorescent staining for Uhrf1 (Red), Pdpn (Green) and DAPI (Blue) in thymus and lymph nodes from *Uhrf1*$^{fl/fl}$ and *Uhrf1*$^{\Delta Col6a1}$ mice. Scale bar represents 50 μm. (**E**) Left, safranin O, fast green and eosin staining of ankle tissue on day 10 after arthritis induction. Scale bar represents 50 μm. Right, relative cartilage area (safranin O positive region) per field in normal (n=3-4), CAIA (n=4-5) and STA (n=7-8) *Uhrf1*$^{fl/fl}$ and *Uhrf1*$^{\Delta Col6a1}$ mice. (**F**) TRAP staining of ankle tissue on day 10 after arthritis induction. Scale bar represents 50 μm. (**G**) Left, binarized image of TRAP staining. Scale bar represents 50 μm. Right, relative TRAP-positive area in the entire ankle area (as in Panel F) in normal (n=3-4), CAIA (n=4-5) and STA (n=7) *Uhrf1*$^{fl/fl}$ and *Uhrf1*$^{\Delta Col6a1}$ mice. (**H**) Representative μCT image of arthritic ankles from *Uhrf1*$^{fl/fl}$ and *Uhrf1*$^{\Delta Col6a1}$ mice. Arrowhead indicates bone damage. Scale bar represents 1 mm. Mean±SD is shown. * and ** indicate $P<0.05$ and $P<0.01$ versus *Uhrf1*$^{fl/fl}$, respectively, by unpaired *t*-test. Data in (B), (E) to (G) were obtained from 3-8 independent experiments.

Data in (C) and (D) were obtained from 3 independent experiments and technically replicated. Data in (H) were obtained from more than 2 independent experiments.

Supplemental Figure 3: Myeloid cell-specific *Uhrf1* depletion does not result in an arthritic phenotype.

(**A**) *Uhrf1* mRNA expression in bone marrow-derived macrophages (BMDM) from $Uhrf1^{fl/fl}$ and $Uhrf1^{\Delta LysM}$ mice (n=3). (**B** and **C**) Development of (B) hind paw thickness and (C) clinical score in $Uhrf1^{fl/fl}$ and $Uhrf1^{\Delta LysM}$ mice after STA induction (n=8-9). (**D** and **E**) Representative (D) safranin O, fast green and eosin staining and (E) TRAP staining in ankle tissue on day 10 after STA induction. Scale bar represents 1 mm. Mean±SD is shown. *N.S.*; not significant versus $Uhrf1^{fl/fl}$. * indicates $P<0.05$ by unpaired *t*-test. All data were obtained from 3-9 independent experiments.

Supplemental Figure 4: GO and pathway analysis in $Uhrf1^{\Delta Col6a1}$ SF.

(**A** and **B**) (A) KEGG pathway analysis and (B) GO analysis among down-regulated genes using DAVID Bioinformatics Resources. Significantly enriched terms are illustrated by gene counts and *P* values. (**C**) Venn diagram to compare genes exhibiting down-regulation

following *Uhrf1* depletion in SF, chondrocytes and hematopoietic stem cells (HSC) based on RNA-seq data from this study and public databases (GSE92641, GSE85450). (**D**) GO biological processes among 105 up-regulated genes with peaks assigned using DAVID Bioinformatics Resources. Enriched pathways are illustrated by gene counts and *P* values. (**E**) Venn diagram for 89 down-regulated genes in $Uhrf1^{\Delta Col6a1}$ SF and 39 genes having Uhrf1-mediated methylated DNA peaks within the gene body. (**F**) KEGG pathway analysis of 39 down-regulated genes with peaks assigned using DAVID Bioinformatics Resources. Significantly enriched pathways are illustrated by gene counts and *P* values. (**G**) Left, flow cytometry analysis to analyze proportion of leukocytes in $Uhrf1^{fl/fl}$ and $Uhrf1^{\Delta Col6a1}$ derived from STA mice on day 4 (n=6-8) and day 10 (n=9-10). Right, quantification of $CD45^+$ $CD4^-$ $Ccr6^+$ cells, $CD45^+$ $CD4^+$ $Ccr6^-$ cells and $CD45^+$ $CD4^-$ $Ccr6^-$ cells among $CD45^+$ cells. Mean±SD is shown. * and ** indicate $P<0.05$ and $P<0.01$, respectively, by ANOVA followed by Tukey's test in (G). Data in (A) to (F) were obtained from combined read data derived from 3 independent experiments. Data in (G) was obtained from 6-10 independent experiments.

Supplemental Figure 5: Correlation between UHRF1 expression level and RA severity.

(**A**) Expression levels of *DNMTs* (*DNMT1, DNMT3A* and *DNMT3B*) mRNA in synovium obtained from OA (n=32) and RA (n=30) patients. (**B**) Spearman's correlation between *UHRF1* mRNA expression in RA synovium and TJC28 (tender joint count 28), SJC28 (swollen joint count 28) and Patient's VAS (visual analogue scale) (n=30). (**C** to **E**) Spearman's correlation between (C) *DNMT1*, (D) *DNMT3A* and (E) *DNMT3B* mRNA expression in RA synovium and DAS28-CRP, CRP, Age, TJC28, SJC28 and Patient's VAS (n=30-31). (**F**) Immunofluorescent staining for UHRF1 (Red), PDPN (Green) and DAPI (Blue) in OA and RA synovium. Scale bar represents 30 μm. (**G**) *UHRF1* and *CCL20* mRNA expression levels in OASF treated with UHRF1 siRNA (n=5). (**H**) Flow cytometry analysis to analyze proportion of Th17 cells ($CD45^+$ $CD4^+$ $CCR6^+$) in OA and RA synovium tissue. Mean±SD is shown. *N.S.*; not significant versus OA by Mann-Whitney U test in (A). * indicates P<0.05 by ANOVA followed by Tukey's test in (G). Data in (A) to (D), (G) and (H) were obtained from 5-32 independent experiments.

Supplemental Figure 6: UHRF1 negatively modulates gene expression of multiple RA-

exacerbating factors in RA synovium.

Among genes involved in maintaining DNA methylation, UHRF1 expression level is elevated in SF, particularly under RA pathogenesis, but not OA. Sufficient levels of UHRF1 can reduce mRNA expression levels of genes that encode multiple RA-exacerbating factors such as RA-related, cytokine-related and anti-apoptosis-related genes by altering DNA methylation. In contrast, insufficient UHRF1 expression levels are associated with progression of RA pathogenesis. These results suggest that UHRF1 stabilization could be a new strategy to develop RA therapeutics.

Supplemental Figure 1

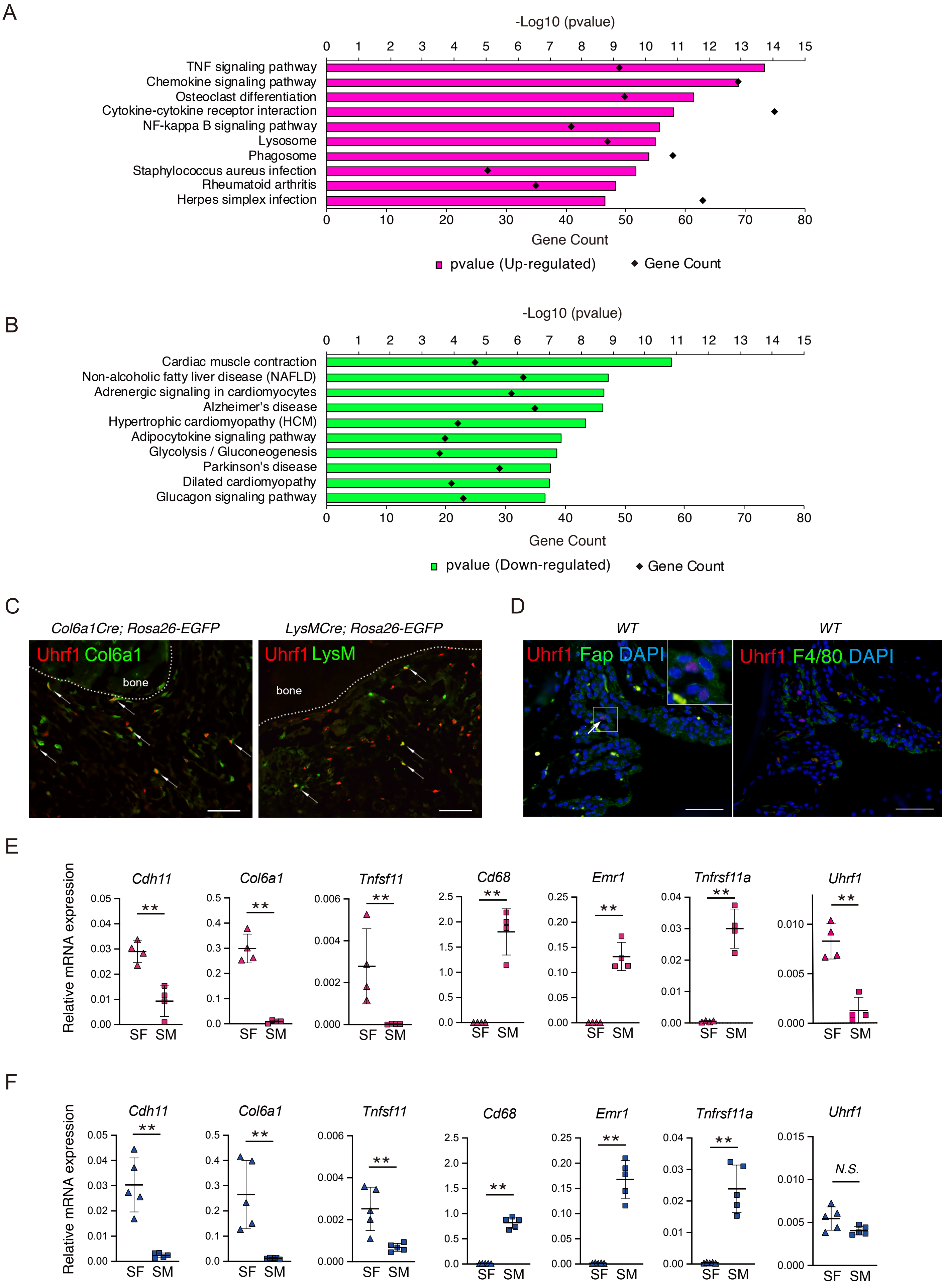

Supplemental Figure 2

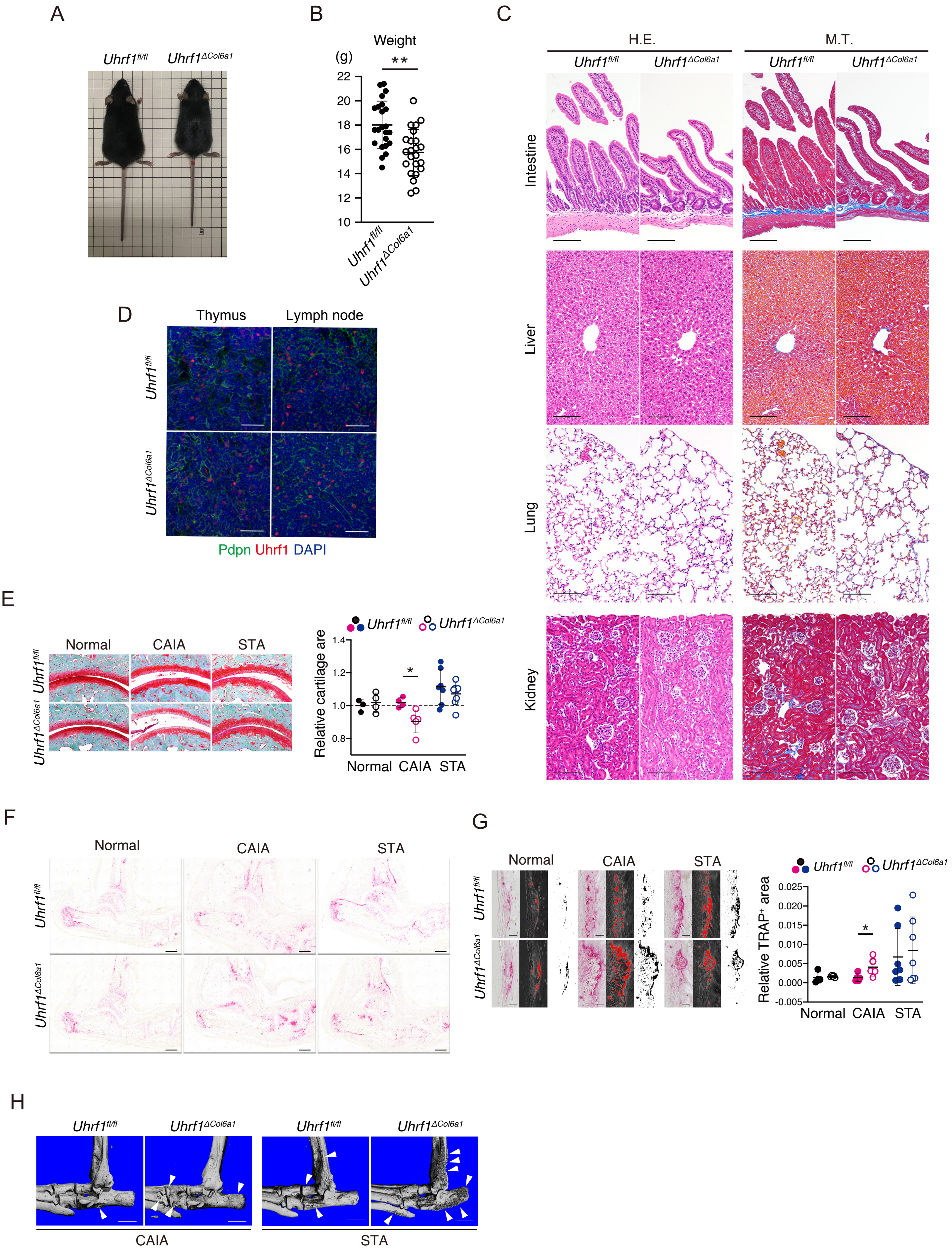

Supplemental Figure 3

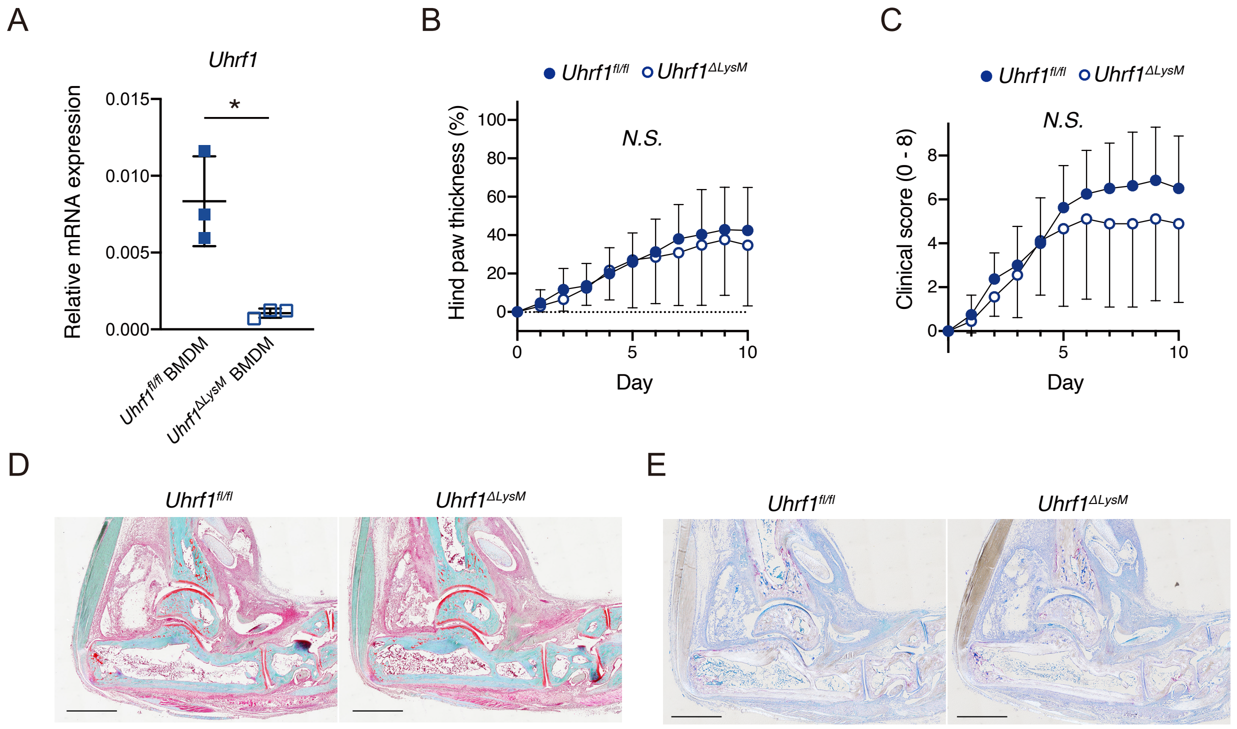

Supplemental Figure 4

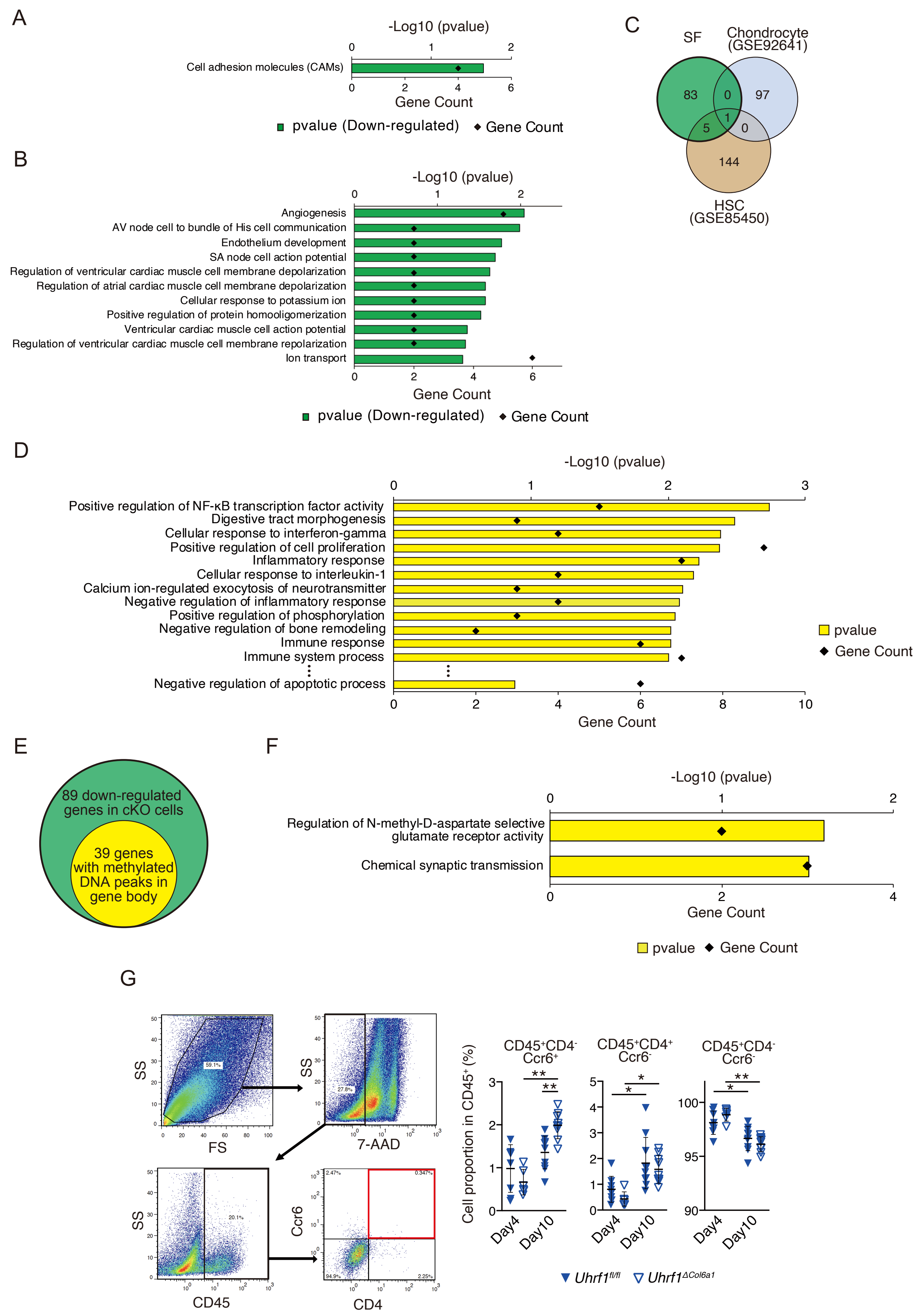

Supplemental Figure 5

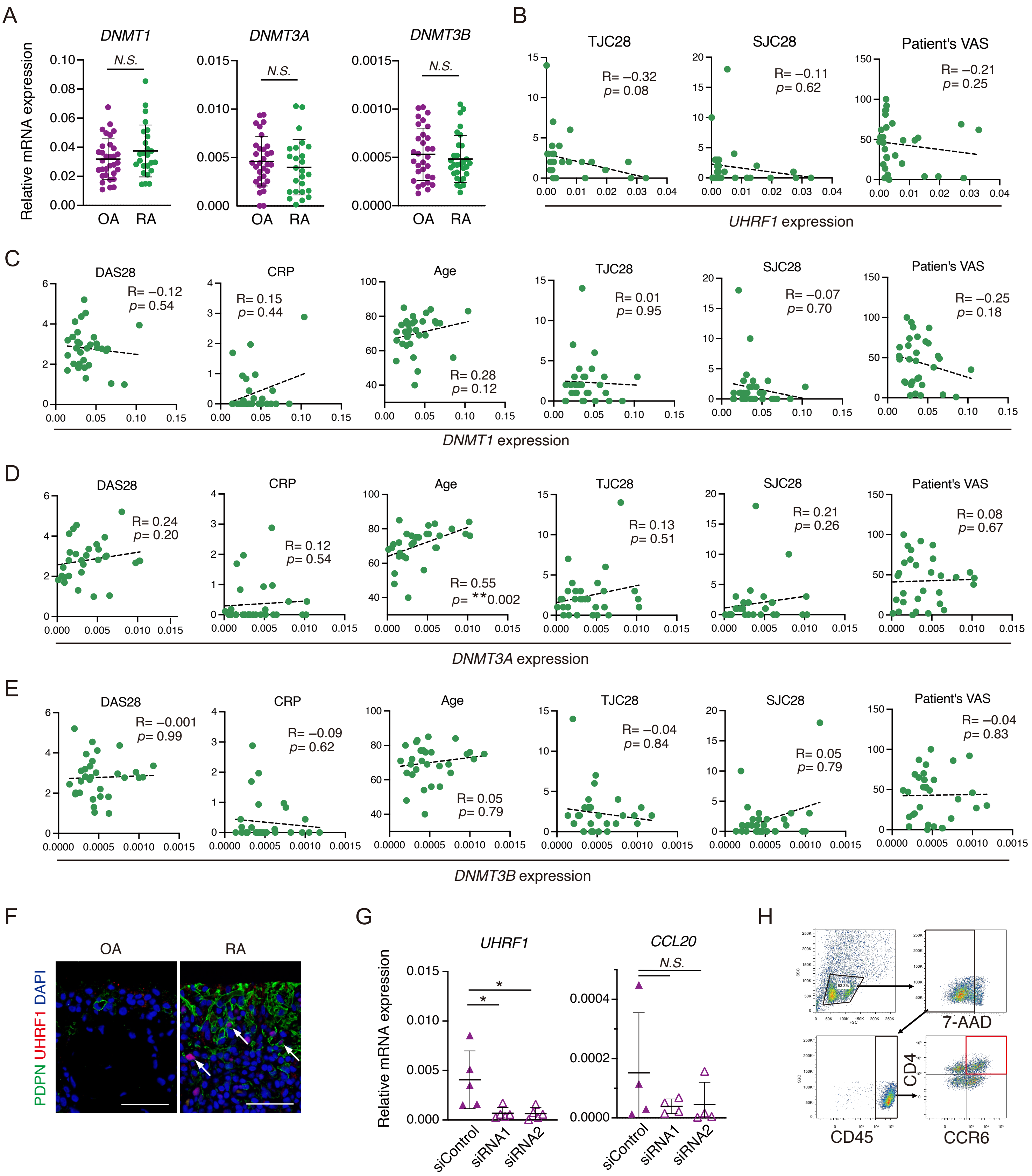

Supplemental Figure 6

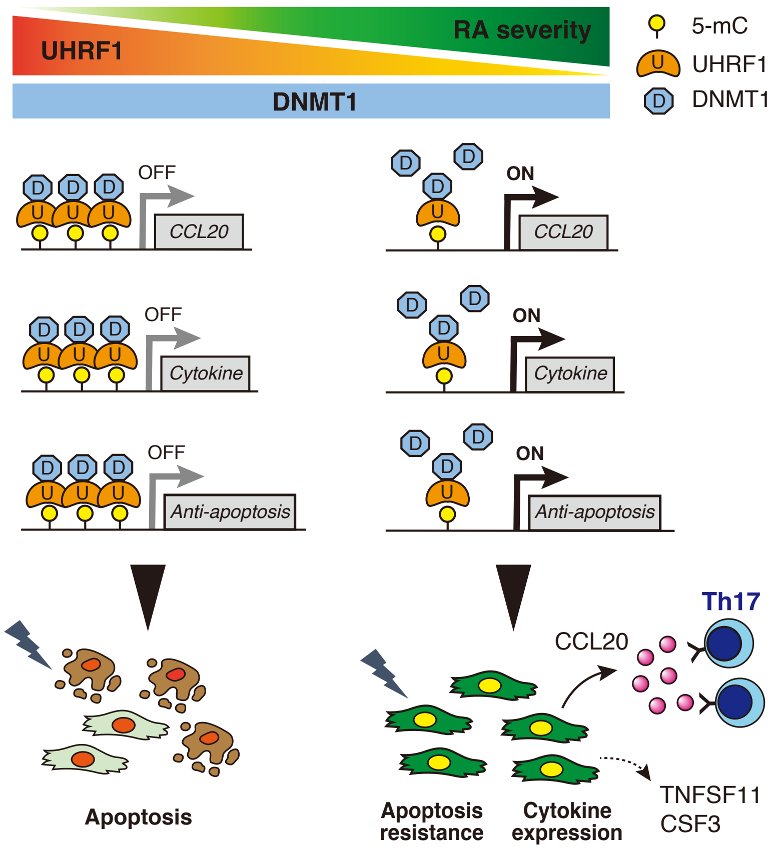

Supplement: Supplemental data [file jci-132-150533-s154.pdf]
